# Ferroptosis in cancer: molecular mechanisms, tumour microenvironment, therapeutic resistance, and clinical translation

**DOI:** 10.1186/s43046-026-00419-x

**Published:** 2026-10-17

**Authors:** Ghisaram Dewasi, Sakshi Jain

**Affiliations:** 1 Indian Council of Medical Research- National Institute of Health Research, Jodhpur, India; 2 Bhupal Nobles’ College of Pharmacy, Udaipur, India

**Keywords:** Ferroptosis, Cancer, Iron metabolism, Lipid peroxidation, Tumour microenvironment, Drug resistance, Immunotherapy, Nanomedicine, Precision oncology, Clinical translation

## Abstract

Ferroptosis is a distinct form of regulated cell death driven by iron-dependent lipid peroxidation and oxidative membrane damage, and it differs fundamentally from apoptosis, necroptosis, and pyroptosis in its molecular mechanisms and execution pathways. Increasing evidence indicates that ferroptosis plays a pivotal role in cancer biology by regulating tumour initiation, progression, metastasis, therapeutic resistance, and interactions within the tumour microenvironment (TME). Dysregulation of iron metabolism, lipid peroxidation, amino acid metabolism, and antioxidant defence systems, including the System Xc⁻–GSH–GPX4, FSP1–CoQ₁₀, GCH1–BH4, and DHODH pathways, collectively determines cellular susceptibility to ferroptosis. Recent studies have further highlighted the contribution of organelle crosstalk, immune cell regulation, and stromal interactions in shaping ferroptotic responses and tumour evolution. In this review, we comprehensively summarise the molecular mechanisms underlying ferroptosis, its regulatory signalling networks, and the major milestones that have advanced the field. We further discuss the multifaceted role of ferroptosis across diverse malignancies, its influence on epithelial–mesenchymal transition, tumour invasion, metastasis, and immune modulation, and its emerging significance in overcoming resistance to chemotherapy, radiotherapy, targeted therapy, and immunotherapy. In addition, we critically evaluate recent progress in ferroptosis-based therapeutic strategies, including nanomedicine, combination therapies, and translational advances, while highlighting current clinical challenges such as tumour heterogeneity, limited pharmacodynamic biomarkers, and safety concerns. Collectively, ferroptosis represents a promising therapeutic vulnerability that bridges fundamental cancer biology with precision oncology. Continued development of biomarker-guided patient stratification, targeted drug delivery systems, and rational combination strategies is expected to accelerate the clinical translation of ferroptosis-based therapies and improve outcomes for patients with refractory and treatment-resistant cancers.

## Introduction

Every living organism undergoes death as an inevitable biological phenomenon, and at the cellular level, this process is fundamental for maintaining tissue integrity and physiological homeostasis [1]. Cell death in mammals is broadly classified into accidental cell death (ACD) and regulated cell death (RCD). Unlike ACD, which results from irreversible physical, chemical, or mechanical insults, RCD is orchestrated through genetically controlled signalling pathways and can be manipulated by pharmacological or genetic interventions [2]. The precise regulation of RCD is indispensable for embryonic development, tissue remodelling, immune surveillance, and the elimination of damaged or dysfunctional cells. Disruption of these tightly regulated death programs enables abnormal cells to escape elimination, facilitating uncontrolled proliferation, prolonged survival, and progressive accumulation of genetic alterations that ultimately contribute to tumour initiation and progression [1, 3]. Consequently, defective regulation of cell death is now recognised as a fundamental hallmark of cancer and a major factor underlying therapeutic resistance, disease recurrence, and poor clinical outcomes.

Effective cancer therapy is fundamentally centred on selectively eliminating malignant cells while preserving the viability and function of normal tissues, thereby maximising therapeutic efficacy and minimising treatment-related toxicity [4]. Among the mechanisms exploited for this purpose, regulated cell death (RCD) has emerged as a critical therapeutic strategy because it enables controlled removal of tumour cells and enhances cancer responsiveness to anticancer agents while reducing collateral damage to healthy tissues [5]. Within this expanding landscape of RCD, ferroptosis, first described by the laboratory of Brent R. Stockwell in 2012, has gained considerable attention as a unique form of cell death driven by iron-dependent lipid peroxidation of cellular membranes [4]. Unlike other well-characterised forms of RCD, including apoptosis, pyroptosis, and necroptosis, ferroptosis is distinguished by its characteristic biochemical, genetic, and morphological features [6, 7]. Importantly, its execution does not rely on the canonical molecular machinery governing these pathways, as ferroptosis proceeds independently of key mediators such as caspases, gasdermin D, and mixed lineage kinase domain-like (MLKL) protein, highlighting its distinct mechanistic identity and therapeutic potential [8].

Morphologically, ferroptosis is distinguished by alterations at both the mitochondrial and plasma membrane levels. Mitochondria typically display reduced volume, increased outer membrane density and marked loss or rupture of cristae, although these features are inducer-dependent and are most evident following cystine deprivation or System Xc⁻ inhibition rather than direct GPX4 inactivation [9, 10]. The most characteristic feature at the cellular level is plasma membrane ballooning: ferroptotic cells progressively swell and adopt a rounded, distended morphology with a clear, organelle-depleted cytoplasmic space before rupture. This reflects an osmotic mechanism of execution, in which accumulating oxidised phospholipids compromise membrane integrity and permit the opening of plasma membrane nanopores; the resulting solute influx drives cell swelling that precedes and is distinct from terminal lysis [11]. Increased membrane tension additionally gates Piezo1 and raises cation permeability, contributing to the execution phase [12], while ESCRT-III-dependent repair opposes pore formation and modulates death kinetics [13]. Importantly, the nucleus remains largely intact, with chromatin condensation, nuclear fragmentation and apoptotic body formation characteristically absent, providing a reliable morphological distinction from apoptosis, while the absence of gasdermin-dependent pore formation and of organised membrane blebbing distinguishes ferroptosis from pyroptosis and necroptosis respectively. Ferroptotic swelling can also propagate to adjacent cells in a lipid peroxide- and iron-dependent manner, generating the wave-like spatial pattern of death characteristic of this pathway [11].At the molecular level, ferroptosis also follows a distinct execution programme. In contrast to classical forms of regulated cell death that rely on well-defined executioner proteins, the terminal effectors responsible for ferroptotic cell death have not yet been conclusively identified. Current evidence indicates that the accumulation of oxidised phospholipids enriched with polyunsaturated fatty acids (PUFA-PLs) is indispensable for ferroptosis; however, the precise molecular events through which these oxidised lipids compromise membrane integrity and irreversibly commit cells to death remain incompletely understood [1, 14]. Overall, ferroptosis is driven by the coordinated interplay of intracellular ferrous iron accumulation, excessive reactive oxygen species generation, impaired antioxidant defences, and uncontrolled lipid peroxidation, which collectively culminate in membrane damage and cell death [15].

Leveraging these distinctive features, a comprehensive panel of biomarkers and functional tests, including pharmacological inhibition, has been assembled to effectively differentiate ferroptosis from other types of RCD, providing suitable tools for investigating its pathophysiological functions [16]. In recent years, modulating ferroptosis to intervene in the occurrence and development of cancer has become a hotspot in etiological research and treatment. Furthermore, ferroptosis is tightly implicated in tumour biology. On the one hand, tumour suppressors have been found to execute part of their tumour-suppression function depending on ferroptosis induction, suggesting that ferroptosis functions as an innate tumour-suppressive mechanism [17, 18]. On the other hand, cancer cells can evolve several mechanisms to evade host ferroptosis, which provides vulnerable targets for ferroptosis-based therapy. Interestingly, mesenchymal and dedifferentiated cancer cells, which typically exhibit resistance to apoptosis and traditional treatment approaches, display a remarkable susceptibility to ferroptosis. Consequently, ferroptosis is recognised as an attractive target for cancer treatments, especially for refractory tumours [19].

Promisingly, a plethora of ferroptosis interventions have shown effectiveness in cancer treatment, even overcoming resistance to traditional therapies [17–19], and ferroptosis is also involved in the tumour-suppressive functions of radiotherapy and immunotherapy [20]. Moreover, combination therapy based on ferroptosis is a highly promising strategy for enhancing the effectiveness of conventional therapies, tackling resistant tumours, and preventing tumour recurrence. However, it is important to note that the role of ferroptosis in tumour suppression depends on the context, as it appears to have paradoxical roles in different stages of some tumours [1, 20]. For instance, ferroptosis induction facilitates the progression of chronic liver diseases to hepatocellular carcinoma [21], while it can restrain established HCC development [22]. Furthermore, a study found that ferroptosis inhibitors can effectively suppress tumour growth if they are administered early when the tumour is sufficiently small [23].

Accordingly, a comprehensive understanding of the molecular basis and biological significance of ferroptosis is essential for translating this form of regulated cell death into effective cancer therapies [20]. With the rapid expansion of ferroptosis research, continuous re-evaluation of emerging discoveries is necessary to bridge fundamental mechanisms with clinical applications. In this review, we provide a comprehensive overview of the historical development and molecular mechanisms governing ferroptosis, highlighting its major regulatory drivers and antioxidant defence systems. We further discuss the multifaceted roles of ferroptosis in tumour biology, its interactions with the tumour microenvironment and immune cells, and the principal signalling pathways that regulate ferroptotic responses in cancer. Finally, we summarise recent advances in ferroptosis-based therapeutic strategies, biomarkers, and clinical translation, while outlining the major challenges and future perspectives for integrating ferroptosis-targeted approaches into precision oncology [8, 24].

## Regulatory mechanisms of ferroptosis

Ferroptosis is driven by disrupted iron homeostasis and unchecked LPO and is mediated by a multilayered network of metabolic regulators, signalling cascades, and organelle interactions [10].

### Iron metabolism

Iron is the most abundant trace element in the human body and plays an essential role in numerous physiological processes, including haem synthesis, iron–sulfur (Fe–S) cluster biogenesis, mitochondrial respiration, and the activity of several iron-dependent enzymes. Under physiological conditions, iron homeostasis is tightly regulated through coordinated uptake, storage, utilisation, and export. Ferric iron (Fe³⁺), bound to transferrin, is internalised via the transferrin receptor (TFR), reduced to ferrous iron (Fe²⁺) by six-transmembrane epithelial antigen of the prostate 3 (STEAP3) within endosomes, while excess intracellular iron is safely stored in ferritin; ferroportin serves as the principal iron exporter to maintain systemic iron balance [1, 25]. Disruption of this homeostatic network increases the intracellular labile Fe²⁺ pool, thereby promoting Fenton and Fenton-like reactions that generate excessive reactive oxygen species (ROS), initiate lipid peroxidation (LPO), and compromise membrane integrity [21]. Mitochondria represent the primary site of intracellular iron utilisation, where Fe²⁺ is transported through voltage-dependent anion channels (VDAC) and mitoferrin (MFRN) to sustain electron transport chain (ETC) function. However, mitochondrial iron overload markedly enhances mitochondrial ROS production, facilitating ferroptotic cell death. In addition, Fe²⁺ serves as an essential cofactor for lipoxygenases (LOXs) and the cytochrome P450 reductase–cytochrome P450 (POR–CYP) system, both of which accelerate phospholipid peroxidation and further amplify ferroptosis [7, 22].

### System Xc^−^ and amino acid metabolism

The System Xc⁻–GSH axis constitutes one of the principal antioxidant defence mechanisms regulating ferroptosis. System Xc⁻, a heterodimer composed of solute carrier family 7 member 11 (SLC7A11/xCT) and solute carrier family 3 member 2 (SLC3A2/CD98), maintains intracellular redox homeostasis by exchanging extracellular cystine for intracellular glutamate. Following cellular uptake, cystine is reduced to cysteine, which serves as the primary substrate for glutathione (GSH) biosynthesis as well as for the production of coenzyme A (CoA) and hydrogen sulfide (H₂S) [26]. In parallel, the transsulfuration pathway provides an additional source of cysteine through methionine metabolism. Newly synthesised GSH cooperates with glutathione peroxidase 4 (GPX4) to neutralise reactive oxygen species (ROS) and phospholipid hydroperoxides, thereby preserving protein function and membrane integrity [27]. The antioxidant activity of GPX4 depends on selenocysteine incorporation via the mevalonate (MVA) pathway, while its cytosolic and mitochondrial isoforms are essential for maintaining cellular redox balance and mitochondrial stability [28]. Glutamine metabolism further influences ferroptosis susceptibility, as glutamine imported through SLC1A5/SLC38A1 is converted into α-ketoglutarate (α-KG), supporting tricarboxylic acid (TCA) cycle activity, lipid metabolism, and iron homeostasis. Under conditions of GSH depletion, glutamine metabolism is redirected towards energy production, resulting in excessive ROS accumulation and enhanced ferroptosis sensitivity [29]. Collectively, the coordinated interaction between the System Xc⁻–GSH–GPX4 axis and amino acid metabolism represents a central determinant of ferroptosis susceptibility and provides important therapeutic opportunities for ferroptosis-inducing agents, including erastin, RSL3, and buthionine sulfoximine (BSO) [30].

### Central carbon metabolism and reducing-equivalent supply

Ferroptosis is increasingly recognised as a metabolic vulnerability rather than solely a redox one, since the antioxidant systems described above are ultimately constrained by the metabolic flux that sustains them. NADPH represents the common currency of this constraint: it regenerates glutathione for GPX4, supplies FSP1-mediated reduction of ubiquinone, and supports the mevalonate pathway and fatty acid synthesis. Consistent with this central position, basal NADPH abundance predicts ferroptosis sensitivity across cancer cell lines, and enzymatic depletion of cytosolic NADPH by the phosphatase MESH1 sensitises cells to ferroptotic death [31, 32]. Reducing-equivalent supply is therefore an upstream determinant of where a tumour sits relative to its ferroptotic threshold.

Mitochondrial and TCA cycle activity feed into this balance in a context-dependent manner. Glutamine imported through SLC1A5/SLC38A1 is converted to α-ketoglutarate to sustain anaplerosis, and glutaminolysis is required for the execution of ferroptosis induced by cystine deprivation, with transferrin-mediated iron delivery acting as the co-requisite signal [33]. Elevated oxidative phosphorylation increases mitochondrial reactive oxygen species and potentiates ferroptosis, whereas loss of TCA cycle enzymes including fumarate hydratase or α-ketoglutarate dehydrogenase components confers resistance to cystine deprivation-induced death. Mitochondria thus act as both amplifiers and buffers of ferroptotic signalling depending on metabolic configuration, a duality also reflected in their organelle-level regulation (discussed below). Energy stress imposes an additional layer of control: AMPK activation under nutrient limitation suppresses ferroptosis by restraining polyunsaturated fatty acid synthesis, such that metabolically stressed tumour cells may paradoxically become more resistant [34].

One-carbon and nucleotide metabolism intersect at the same nodes. The methionine cycle and transsulfuration pathway provide an alternative cysteine supply that sustains glutathione synthesis when System Xc⁻ is inhibited, while serine-derived one-carbon flux contributes to NADPH regeneration; the dependency imposed by a given ferroptosis inducer therefore differs according to which of these routes is available [35]. Nucleotide metabolism is linked through DHODH, which couples de novo pyrimidine synthesis to mitochondrial CoQ₁₀ reduction [36].

Collectively, these observations indicate that metabolic plasticity, the capacity to reroute carbon and reducing equivalents between these pathways is itself a determinant of ferroptosis sensitivity, and a plausible mechanism by which tumours adapt to ferroptosis-inducing therapy without altering expression of the canonical defence genes.

### Lipid metabolism and alternative pathways

Lipid peroxidation (LPO) is the defining biochemical event underlying ferroptosis, with polyunsaturated fatty acids (PUFAs) serving as its principal substrates. Among these, arachidonic acid (AA) and adrenic acid (ADA) are first activated by acyl-CoA synthetase long-chain family member 4 (ACSL4) and subsequently incorporated into membrane phospholipids by lysophosphatidylcholine acyltransferase 3 (LPCAT3), rendering them highly susceptible to oxidative damage [37]. Oxidation of these PUFA-containing phospholipids occurs through both non-enzymatic Fenton chemistry and enzymatic pathways mediated by lipoxygenases (LOXs) and cytochrome P450 oxidoreductase (POR), leading to the generation of phospholipid hydroperoxides (PLOOH). Secondary lipid peroxidation products, including malondialdehyde (MDA) and 4-hydroxynonenal (4-HNE), further propagate oxidative stress and inflammatory signalling, thereby amplifying ferroptotic damage [38]. This process occurs across multiple membrane-bound organelles, while lipid droplets (LDs) serve as dynamic reservoirs that release PUFAs during oxidative stress to sustain lipid peroxidation. Progressive accumulation of membrane lipid damage ultimately culminates in plasma membrane disruption, representing the irreversible execution phase of ferroptosis [16].

Beyond the System Xc⁻–GSH–GPX4 axis, cells employ multiple antioxidant defence and membrane repair mechanisms to counteract excessive lipid peroxidation and maintain ferroptosis resistance once lipid radicals accumulate beyond a critical threshold. Ferroptosis suppressor protein 1 (FSP1), initially localised to mitochondria, undergoes N-terminal myristoylation and subsequently translocates to the plasma membrane and lipid droplets (LDs), where it utilises NAD(P)H to reduce ubiquinone (CoQ₁₀) and vitamin K into potent radical-trapping antioxidants that preserve membrane integrity [39, 40]. CoQ₁₀, generated through the mevalonate (MVA) pathway, not only supports mitochondrial respiration but also functions as an effective scavenger of reactive oxygen species. Within mitochondria, dihydroorotate dehydrogenase (DHODH) provides an additional defence mechanism by transferring electrons to CoQ₁₀ during pyrimidine biosynthesis, thereby suppressing mitochondrial ferroptosis. Similarly, GTP cyclohydrolase 1 (GCH1) generates tetrahydrobiopterin (BH4), which limits lipid peroxidation and promotes CoQ₁₀ biosynthesis. Cellular resistance to ferroptosis is further reinforced through membrane repair mechanisms, including endosomal sorting complexes required for transport (ESCRT)-III, which repair membrane lesions in response to calcium signalling, and group VIA calcium-independent phospholipase A2 (iPLA2β), which selectively removes oxidised phospholipids [41]. Membrane lipid composition also determines ferroptosis susceptibility, as monounsaturated fatty acids (MUFAs) and saturated fatty acids (SFAs) reduce phospholipid vulnerability by competing with PUFAs for membrane incorporation, whereas deuterated PUFAs (D-PUFAs) resist lipid peroxidation owing to their enhanced chemical stability, thereby inhibiting ferroptosis [42] (Fig. 1).

**Fig. 1:**
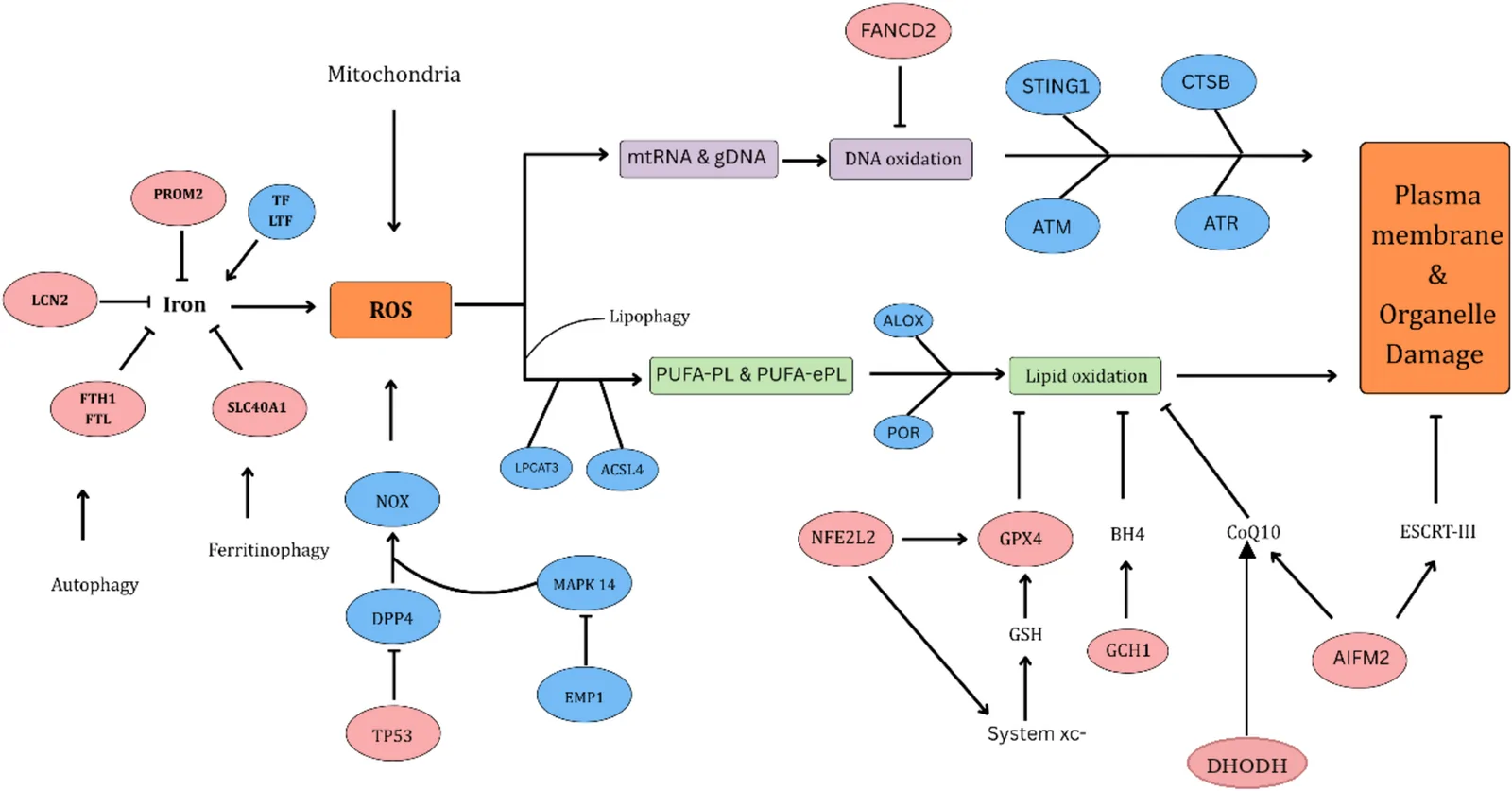
Integrated molecular mechanisms of ferroptosis highlighting iron metabolism, ROS accumulation, lipid peroxidation, and antioxidant regulatory pathways. Iron homeostasis is regulated by iron transport, storage, and ferritinophagy, leading to intracellular iron accumulation and reactive oxygen species (ROS) generation. ROS promotes DNA oxidation and phospholipid peroxidation through ACSL4, LPCAT3, ALOX, and POR, ultimately resulting in plasma membrane and organelle damage. Ferroptosis is counteracted by multiple antioxidant defence pathways, including the System Xc⁻–GSH–GPX4 axis, GCH1–BH4 pathway, AIFM2/FSP1–CoQ₁₀ system, and ESCRT-III-mediated membrane repair. Additional regulators, including TP53, DPP4, NFE2L2 (NRF2), FANCD2, STING1, ATM, ATR, and CTSB, further modulate ferroptosis sensitivity by influencing redox homeostasis, lipid peroxidation, DNA damage responses, and membrane integrity

#### Architecture of the ferroptosis defence network: parallel arms under conditional hierarchy

The four systems described above are usually presented separately, but their interaction under therapeutic pressure is more informative than their individual biochemistry. Two models have been proposed: a hierarchical network with GPX4 dominant and the remainder as subordinate backups, or a parallel system in which each arm contributes interchangeably. Evidence supports neither in pure form, indicating instead a spatially compartmentalised network of partially redundant arms under conditional, insult-dependent hierarchy.

Against strict hierarchy, FSP1 acts alongside rather than downstream of GPX4, anchoring via N-terminal myristoylation to the plasma membrane and lipid droplets, and sustains viability even upon complete GPX4 ablation [43, 44]. Against simple parallelism, NQO1 failed to restore resistance in FSP1-null cells even when targeted to the plasma membrane, despite comparable catalytic capability [43]. Dependency is instead conditional: BH4 becomes essential under GPX4 inhibition, but not cystine deprivation [35], and compartmentalisation explains this specificity: GPX4 acts in the cytosol and mitochondria, FSP1 at non-mitochondrial membranes, and DHODH within the inner mitochondrial membrane, where its inactivation is disproportionately lethal in GPX4-low tumours [36]. A hierarchical layer is imposed transcriptionally, since NRF2 coordinates multiple arms simultaneously; KEAP1-inactive lung cancers upregulate a CoQ–FSP1 axis driving ferroptosis and radiation resistance [45].

This architecture explains how resistance emerges. Because arms are independently regulated, suppressing one unmasks dependency on another rather than reducing total buffering proportionately: persister cells surviving partially lethal GPX4 inhibition become selectively sensitised to FSP1 inhibition [46]. Monotherapy against any single arm should therefore select for cells in which an alternative arm predominates, plausibly contributing to the limited durability of single-agent ferroptosis induction. Combination design follows: GPX4 and FSP1 co-inhibition synergies across multiple cancers, and FSP1 inhibitors have been developed explicitly for combination rather than monotherapy [47]. Critically, the optimal partner is context-specific: GPX4-low or mitochondrially biased tumours favour DHODH co-targeting, NRF2/KEAP1-altered tumours favour FSP1 co-targeting, and hormone-receptor-driven tumours may require endocrine blockade [36, 45, 48].

## Organelle crosstalk in ferroptosis

Ferroptosis is regulated by multiple organelles, including mitochondria, lysosomes, endoplasmic reticulum (ER), Golgi apparatus, and peroxisomes. These organelles coordinate iron metabolism, LPO, and antioxidant defence through intrinsic pathways and crosstalk, collectively determining cell fate.

### Mitochondria

Mitochondria are central regulators of ferroptosis owing to their essential roles in iron metabolism, lipid metabolism, amino acid utilisation, energy production, and mitophagy [49]. Mitochondrial iron homeostasis is maintained through transporters including MFRN1/2, VDAC, and piezo1, whose activity promotes intracellular Fe²⁺ accumulation and mitochondrial reactive oxygen species (mtROS) generation, whereas mitochondrial ferritin (FtMt) and ferritinophagy dynamically regulate iron storage and release, thereby influencing ferroptosis susceptibility. Mitochondrial lipid composition also contributes to ferroptotic sensitivity, as PC-PUFA₂ and cardiolipin (CL) facilitate lipid peroxidation, while 7-dehydrocholesterol (7-DHC) limits phospholipid radical propagation and protects mitochondrial membranes [50, 51]. Metabolic status further determines ferroptosis sensitivity; SLC25A1 enhances acetyl-CoA production and ferroptosis resistance, whereas cysteine deprivation or elevated oxidative phosphorylation (OXPHOS) induces mitochondrial hyperpolarisation and potentiates ferroptotic signalling. In addition, mitochondrial DNA (mtDNA) damage activates the cGAS–STING pathway, while mitochondrial Ca²⁺ overload mediated by MICU1 or matrix acidification further accelerates ferroptosis; conversely, inhibition of the mitochondrial calcium uniporter (MCU) attenuates this process. Mitophagy also exerts a context-dependent role, with moderate mitophagy preserving mitochondrial quality, whereas excessive mitophagy or radiation-induced mitochondrial damage releases iron and fatty acids that further amplify ferroptosis. Therapeutically, mitochondrial-targeted approaches, including the combination of arsenic trioxide (ATO) and sulfasalazine, have demonstrated synergistic induction of mitochondrial ferroptosis and represent promising strategies for future clinical translation [1, 52, 53].

### Lysosomes

Emerging evidence suggests that ferroptosis can also be regarded as a lysosome-dependent form of regulated cell death, highlighting the pivotal contribution of lysosomal function to iron homeostasis and lipid peroxidation [54]. Pharmacological inhibition of lysosomal activity through blockade of cathepsins or vacuolar-type H⁺-ATPase markedly suppresses erastin-induced ferroptosis, whereas activation of lysosomes by quercetin accelerates ferritin degradation and promotes ferroptotic cell death, underscoring the importance of lysosome-mediated ferritinophagy [55]. During ferritinophagy, nuclear receptor coactivator 4 (NCOA4) delivers ferritin to lysosomes for degradation, thereby releasing intracellular free iron. Loss of NCOA4 impairs ferritin degradation, reduces the intracellular labile iron pool, and consequently diminishes ferroptosis sensitivity [56]. Ferroptosis induction is accompanied by autophagy activation, resulting in ferritin degradation, increased labile iron accumulation, and excessive reactive oxygen species (ROS) production. Conversely, inhibition of autophagy or NCOA4 prevents ferritinophagy, suppresses iron and ROS accumulation, and protects cells from ferroptosis. In addition, lipid ROS accumulate within lysosomes, and inhibition of lysosomal activity reduces lipid ROS generation and intracellular iron availability, indicating that lysosomes regulate ferroptosis by controlling cellular iron equilibrium and oxidative stress [7, 57, 58].

Beyond ferritinophagy, lysosomal cathepsins, particularly cathepsin B, have emerged as important mediators of ferroptotic cell death. Translocation of cathepsin B from lysosomes to the nucleus promotes DNA damage and STING1-dependent ferroptosis, whereas pharmacological inhibition of cathepsin B preserves membrane integrity and limits chromatin degradation independently of GPX4 [19, 59]. Furthermore, lysosomes function as a major labile iron reservoir by recycling endogenous iron stores such as ferritin, thereby directly influencing cellular susceptibility to ferroptotic stimuli [60]. The autophagy adaptor SQSTM1/p62 further enhances ferroptosis by mediating lysosomal degradation of the iron exporter FPN1, promoting intracellular iron accumulation required for ferroptotic execution [61].

### Golgi apparatus

The Golgi apparatus (GA) is a membrane-bound organelle primarily responsible for protein processing, trafficking, and secretion, while also functioning as a cellular stress sensor, regulator of lipid and protein modification, mitotic checkpoint, and contributor to malignant transformation [62]. Under pathological conditions the normally continuous Golgi ribbon becomes fragmented, a change associated with cellular dysfunction and death and recognised as a hallmark of apoptotic progression, in which several Golgi structural proteins act as caspase substrates [63–65]. More recently the Golgi has been identified as a regulator of ferroptosis: disruption of Golgi homeostasis by stress-inducing agents, such as brefeldin A, promotes lipid peroxidation and ferroptotic cell death [66].

Golgi stress represents an adaptive response that is activated when the functional capacity of the Golgi apparatus becomes insufficient to meet cellular demand. In mammalian cells, this response involves multiple signalling pathways, including TFE3, heat shock protein 47 (HSP47), CREB3, E26 transformation-specific (ETS), proteoglycan, and mucin pathways, several of which are closely linked to apoptotic regulation [67]. Emerging studies further demonstrate that Golgi stress also contributes to ferroptosis. In HeLa cells, Golgi stress-inducing agents trigger ferroptosis, whereas overexpression of SLC7A11 or GPX4, as well as depletion of ACSL4, effectively suppresses this response. Consistently, pharmacological ferroptosis inhibitors not only prevent cell death but also preserve Golgi architecture and restore protein secretion following Golgi stress, highlighting the critical role of Golgi homeostasis in regulating ferroptotic susceptibility [42].

### Endoplasmic reticulum

The endoplasmic reticulum (ER) is highly enriched with polyunsaturated fatty acids (PUFAs) and represents one of the principal intracellular sites of lipid peroxidation during ferroptosis. ER stress (ERS) contributes to ferroptotic susceptibility by regulating iron metabolism through the induction of iron regulatory proteins and ferritinophagy. In addition, the ER stress-responsive transcription factor ATF4 exerts context-dependent effects on ferroptosis, while ERS stabilises ACSL4 by suppressing membrane-associated ring-CH-type finger 6 (MARCH6), thereby facilitating phospholipid peroxidation [68]. The close functional coupling between the ER and mitochondria further amplifies ferroptosis, as Ca²⁺ transfer through the inositol 1,4,5-trisphosphate receptor (IP3R)–glucose-regulated protein 75 (GRP75)–VDAC1–MCU signalling axis enhances mitochondrial oxidative stress. In contrast, membrane-spanning 4-domains subfamily A member 15 (MS4A15) lowers ER calcium stores and reduces ferroptosis sensitivity, whereas accumulation of Cu²⁺ and Ga³⁺ within the ER further aggravates ferroptotic damage [68]. Recent studies have also identified the ER membrane as a major platform for lipid peroxidation, where activation of the unfolded protein response together with systemic cystine deficiency markedly influences ferroptosis sensitivity [69]. Notably, aggregation of polyunsaturated phospholipids increases ER membrane rigidity, providing a structural hallmark of ferroptotic vulnerability [70]. Moreover, disruption of the transsulfuration pathway abolishes the cytoprotective interaction between Golgi stress and erastin-induced ferroptosis, further emphasising the coordinated crosstalk between intracellular organelles during ferroptotic regulation [71].

Lipid droplets (LDs), which originate from the ER, function as intracellular reservoirs that protect cells against ferroptosis by esterifying excess PUFAs and limiting lipid peroxidation. Loss of LD biogenesis or degradation of the LD-associated protein perilipin 2 (PLIN2) compromises this protective mechanism and increases ferroptosis susceptibility. Conversely, many drug-resistant tumour cells accumulate LDs to evade ferroptotic cell death, whereas LD-targeted photosensitisers restore ferroptosis sensitivity and suppress tumour progression [1].

## Major milestones in ferroptosis research

Although the term ferroptosis was formally introduced only in 2012, the scientific foundations of this form of regulated cell death were established over several decades of research [15]. Iron-induced toxicity was documented as early as 1908 [72], and studies from 1955 established that cystine is indispensable for the viability of cultured mammalian cells [30]. A major advance came in 1977, when Bannai and colleagues reported that cystine deprivation induced cell death through glutathione (GSH) depletion, preventable by vitamin E [73]; their subsequent work identified System Xc⁻ as the cystine–glutamate antiporter. The characterisation of glutathione peroxidase 4 (GPX4) as a GSH-dependent selenoprotein in 1982 demonstrated its essential role in preventing membrane lipid peroxidation [18], and by 1989 glutamate-induced cytotoxicity was attributed to blockade of cystine uptake with consequent GSH depletion and oxidative death [1]. The concept of oxytosis, proposed in 2001 for oxidative stress-induced non-apoptotic neuronal death, shares reactive oxygen species (ROS) generation, GSH depletion, ALOX activation and iron-chelator sensitivity with ferroptosis, but is distinguished by cGMP-gated channel activation, mitochondrial swelling and DNA fragmentation [1, 10].

The modern era of ferroptosis research began with high-throughput small-molecule screening designed to identify compounds selectively targeting oncogenic RAS-driven cancers. In 2003, erastin emerged as the first small molecule capable of inducing a non-apoptotic form of cell death in ST- and RAS-G12V-dependent tumour cells by inhibiting System Xc⁻ and influencing the RAS/BRAF/MEK/MAPK signalling pathway as well as voltage-dependent anion channels (VDACs), thereby promoting oxidative stress and mitochondrial dysfunction [1, 74]. This discovery was followed in 2008 by the identification of RSL3, another small molecule that triggered an iron-dependent mode of cell death through direct inhibition of GPX4 [75]. During the same period, GPX4 inactivation was recognised to induce a non-apoptotic cell death that could be prevented by α-tocopherol and ALOX12/15 inhibitors, highlighting lipid peroxidation as a central event in ferroptosis. These findings culminated in 2012, when Stockwell and colleagues formally defined ferroptosis as an iron-dependent, lipid peroxidation-driven form of regulated cell death, distinguished from previously described oxidative death pathways by its morphological, biochemical and genetic features [76, 77]. The same period established erastin as an inhibitor of System Xc⁻-mediated cystine uptake and ferrostatin-1 as the first potent ferroptosis inhibitor [1, 18, 78–80].

Subsequent years witnessed rapid advances in elucidating the molecular machinery governing ferroptosis. In 2014, GPX4 was confirmed as the central regulator of both erastin- and RSL3-induced ferroptosis, with RSL3 directly targeting the catalytic activity of GPX4 to promote phospholipid peroxidation. During the same period, liproxstatin-1 was identified as a potent ferroptosis inhibitor capable of rescuing GPX4-deficient cells [9, 81]. In 2015, genome-wide insertional mutagenesis in haploid KBM7 cells revealed ACSL4 and LPCAT3 as indispensable determinants of ferroptosis sensitivity, while p53 was shown to enhance ferroptosis by repressing SLC7A11 expression and limiting cystine uptake [1, 82]. In 2016, ferroptosis was linked to ALOX-mediated PUFA oxidation through a PHKG2-dependent iron pool, the catalytic selenocysteine of GPX4 was shown to be essential, and FIN56 was reported to both degrade GPX4 and deplete coenzyme Q₁₀ via the mevalonate pathway [1]. ACSL4 was identified in 2017 as an essential executioner catalysing incorporation of arachidonic and adrenic acid into phosphatidylethanolamines [83], and selenium utilisation was shown to be required for GPX4 activity in 2018. Two landmark findings followed in 2019: CD8⁺ T cells were shown to induce tumour ferroptosis during immunotherapy, and independent genetic screens identified ferroptosis suppressor protein 1 (FSP1; formerly AIFM2) as a GPX4-independent defence mechanism [1, 39, 84].

Two independent studies published in 2020 identified GTP cyclohydrolase 1 (GCH1) as a GPX4-independent suppressor of ferroptosis. GCH1 limits ferroptosis by generating the lipophilic antioxidant tetrahydrobiopterin (BH4), which directly inhibits lipid peroxidation, while simultaneously increasing the abundance of coenzyme Q10 (CoQ₁₀) to reinforce cellular antioxidant defence [85–87]. In the same year, Zou et al. identified peroxisomes as critical regulators of ferroptosis through the synthesis of polyunsaturated ether phospholipids (PUFA-ePLs), which serve as substrates for lipid peroxidation [88]. Cyst(e)inase was shown to induce ferroptosis in pancreatic ductal adenocarcinoma by depleting cysteine, whereas the lymphatic microenvironment was found to protect metastasising melanoma cells from ferroptosis [89, 90]. In 2021, dihydroorotate dehydrogenase (DHODH) was identified as a mitochondrial defence system that suppresses ferroptosis through the CoQ₁₀ pathway, while DHODH inhibition was shown to enhance ferroptosis sensitivity, particularly in cooperation with FSP1 inhibition [91]. During 2022, spontaneous ferroptosis of polymorphonuclear myeloid-derived suppressor cells (PMN-MDSCs) was recognised as a critical mechanism contributing to tumour-associated immune suppression, extending the role of ferroptosis to immune regulation within the tumour microenvironment. In addition, FSP1 was demonstrated to reduce vitamin K to its hydroquinone form through a non-canonical cycle, providing an alternative antioxidant system against lipid peroxidation [92]. Subsequent studies in 2023 further revealed that FSP1-dependent phase separation is essential for efficient ferroptosis suppression [1, 93]. More recently, Zhao et al. demonstrated that reduced protein synthesis increases the susceptibility of haematopoietic stem cells to ferroptosis, while genome-wide CRISPR activation screening identified MBOAT1 and MBOAT2 as sex hormone-regulated ferroptosis defence factors capable of protecting cancer cells independently of both GPX4 and FSP1 [1]. In 2025, a major conceptual advance demonstrated that ferroptosis is not restricted to individual cells but can propagate between neighbouring cells. Using an optogenetic GPX4-degradation system, researchers showed that lipid peroxidation and ferroptotic cell death spread through closely adjacent plasma membrane contacts in an α-catenin-dependent and extracellular iron-dependent manner, establishing ferroptosis as a propagative form of regulated cell death and providing new insights into tissue injury, tumour progression and therapeutic targeting [94]. Collectively, these discoveries have transformed ferroptosis into a field encompassing GPX4-independent defence systems, organelle-specific regulation, immune modulation, intercellular communication and translational opportunity (Fig. 2).

**Fig. 2:**
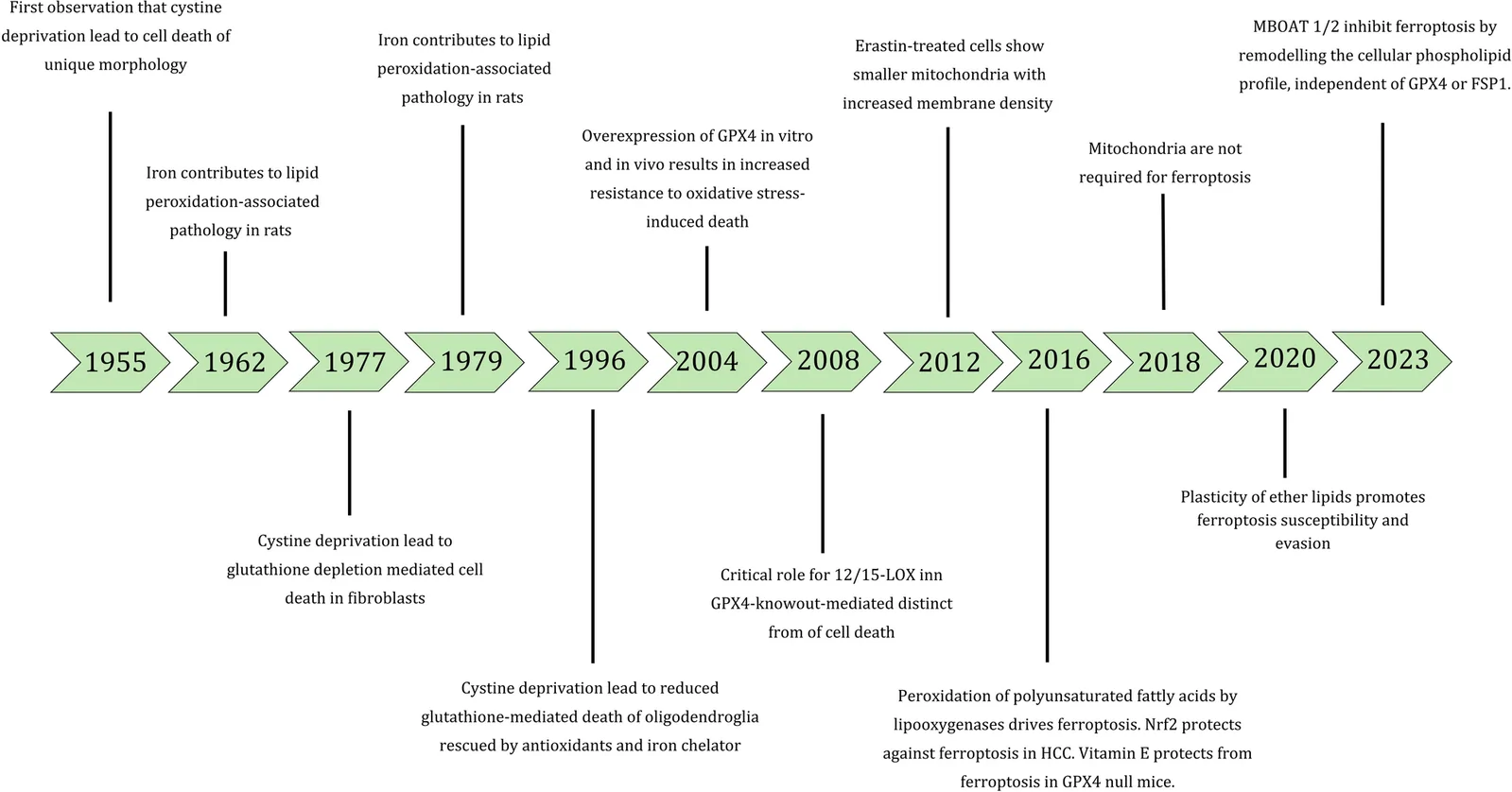
Major milestones in ferroptosis research from 1955 to 2025. The timeline highlights landmark discoveries that have shaped the understanding of ferroptosis, beginning with early observations of cystine deprivation-induced cell death and iron-dependent lipid peroxidation, followed by the identification of GPX4 as a central regulator, the formal definition of ferroptosis in 2012, and subsequent advances in lipid metabolism, mitochondrial biology, membrane repair, and phospholipid remodelling. Together, these discoveries have established ferroptosis as a key regulator of cancer biology and a promising therapeutic target

## Cellular ferroptosis competence: an integrated framework for ferroptosis sensitivity in cancer

Although the preceding sections describe individual regulators of ferroptosis, the susceptibility of a given cancer cell is not determined by any single pathway. Ferroptosis is defined by a common terminal biochemistry iron-dependent peroxidation of polyunsaturated phospholipids beyond the capacity of the cell to detoxify or repair the resulting damage - yet sensitivity to this process varies markedly between tissues, tumour types, and even between subclones of the same tumour [95, 96]. What differs between a ferroptosis-sensitive and a ferroptosis-resistant cell is therefore not the presence of the ferroptotic machinery, but the metabolic and lipidomic context that determines how readily lipid peroxidation can be initiated, amplified, and contained [95].

We therefore propose that ferroptosis susceptibility is most usefully conceptualised as cellular ferroptosis competence: a functional threshold established by the balance between pro-ferroptotic pressure and anti-ferroptotic buffering capacity. Pro-ferroptotic pressure is set by the size of the labile iron pool, the abundance of peroxidisable PUFA-containing phospholipids generated through the ACSL4–LPCAT3 axis, and the prevailing oxidative and metabolic stress imposed by the microenvironment. Anti-ferroptotic buffering is provided by the partially redundant defence systems described above - System Xc⁻–GSH–GPX4, FSP1–CoQ₁₀, GCH1–BH4, and mitochondrial DHODH - together with lipid-remodelling programmes that reduce membrane oxidisability and membrane-repair mechanisms such as ESCRT-III and iPLA2β. A cell becomes ferroptosis-competent when pro-ferroptotic pressure approaches or exceeds this buffering capacity, rather than when any individual gene is gained or lost. Notably, these defence systems are not interchangeable: metabolic screens have shown that GPX4 inhibition and cystine deprivation impose distinct dependencies, with BH4 biosynthesis becoming essential under GPX4 inhibition but not under cystine starvation [35]. Ferroptosis competence is therefore better described as a position along a continuum than as a binary state.

Four classes of determinant position a tumour along this continuum. First, membrane lipid architecture. Selective incorporation of omega-6 PUFAs into phosphatidylethanolamine species by ACSL4 and LPCAT3 generates the oxidisable substrate pool, whereas monounsaturated fatty acid remodelling driven by SCD1 and ACSL3, and phospholipid remodelling by MBOAT1/2, reduce membrane oxidisability and confer resistance independently of GPX4 and FSP1 [48, 97]. Sterol intermediates, including 7-dehydrocholesterol, provide a further layer of lipid-intrinsic radical-trapping protection [98]. Second, antioxidant and metabolic reserve. Cysteine and glutamine availability, NADPH regenerative capacity, selenium supply for GPX4 maturation, and KEAP1/NRF2-driven transcriptional rewiring collectively determine buffering capacity, and tumour metabolic reprogramming may either increase pro-ferroptotic pressure or reinforce resistance depending on which of these nodes dominates [35, 95]. Third, lineage and genotype. Ferroptosis competence is in part hard-wired by cell of origin and driver genotype. Clear-cell carcinomas of the kidney and ovary display an intrinsic GPX4 dependency arising from HIF-2α-driven, HILPDA-dependent enrichment of polyunsaturated lipids, a vulnerability that transcends genetic landscape [99]. Conversely, MBOAT1 and MBOAT2 are transcriptionally driven by oestrogen and androgen receptor signalling respectively, rendering ER-positive breast and AR-positive prostate cancers constitutively buffered against ferroptosis unless hormone signalling is blocked [97]. Fourth, cell state and plasticity. Cancer cells occupying a high-mesenchymal, ZEB1-high state acquire a dependency on the GPX4 lipid-peroxidase pathway, a phenotype observed across EMT in epithelial carcinomas, TGF-β-driven therapy resistance in melanoma, neuroendocrine transdifferentiation in prostate cancer, and sarcomas fixed in a mesenchymal state by their cell of origin [100]. ZEB1 itself links plasticity to ferroptosis sensitivity by reprogramming lipogenic enzyme expression and phospholipid composition [101]. Drug-tolerant persister cells arising from diverse tumours and treatments converge on this same GPX4-dependent state, such that loss of GPX4 function selectively kills persisters and prevents tumour relapse in murine models [102]. Multi-stage dedifferentiation in melanoma similarly defines subtypes with differential vulnerability to iron-dependent oxidative stress [103].

Two implications follow from this framework, both directly relevant to how the tumour-type-specific literature summarised below should be interpreted. First, ferroptosis competence is acquired and lost dynamically rather than fixed. Tumours may move towards competence during EMT, dedifferentiation, hypoxic adaptation, or entry into a drug-tolerant persister state, and away from competence through MUFA remodelling, NRF2 activation, lipid droplet sequestration, or hormone-receptor-driven phospholipid remodelling under therapeutic pressure. This explains why apoptosis-refractory, treatment-resistant residual disease is often the state in which ferroptosis vulnerability is greatest, and why ferroptosis induction is conceptually attractive precisely where conventional cytotoxic therapy fails. Second, because competence is a composite property, no single biomarker will predict it. Expression of SLC7A11, GPX4, or ACSL4 in isolation captures only one axis of a multi-axis balance, which is a plausible contributor to the limited predictive performance of single-gene ferroptosis markers to date. Prospective stratification will likely require integrated readouts spanning lipidomic composition, antioxidant-pathway status, and cell-state signature, a point returned to in the discussion of clinical translation below.

The tumour-type-specific observations described in the following section should therefore be read not as evidence that particular cancers are intrinsically ferroptosis-sensitive or resistant, but as illustrations of where individual tumour lineages and states tend to sit along this shared continuum.

## Role of ferroptosis in cancer

Studies have demonstrated that ferroptosis plays an essential role in cancer. However, different cancer cells vary in their sensitivity to ferroptosis. These differences may be caused by differences in the pathological environment and metabolic status of cancer cells. Here, we discuss the role of ferroptosis in various cancers.

### Ovarian Cancer

Iron handling is markedly altered in ovarian cancer, and this appears to underlie much of its ferroptotic behaviour [28]. Ovarian cancer cells exhibit marked alterations in iron metabolism, where upregulation of transferrin receptor (TFRC) or downregulation of ferroportin (FPN) promotes intracellular iron accumulation, thereby increasing susceptibility to ferroptosis. Consistently, elevated levels of reactive oxygen species (ROS) have been observed across multiple ovarian cancer cell lines, reflecting enhanced oxidative stress and a heightened propensity for ferroptotic cell death. Beyond its involvement in tumour cell survival, ferroptosis also has important prognostic implications. You et al. developed a ferroptosis-related gene signature demonstrating that ferroptosis influences ovarian cancer progression by modulating tumour metastasis and reshaping the tumour immune microenvironment [104]. Likewise, Li et al. established a prognostic model using eight ferroptosis-associated genes from the Gene Expression Omnibus (GEO) database, highlighting the predictive value of ferroptosis-related biomarkers in ovarian cancer. Among these, increased expression of cytochrome b reductase 1 (CYBRD1) was significantly associated with poor clinical outcomes owing to its involvement in ferroptosis-regulatory pathways [105].

Therapeutic targeting follows from these observations. ACSL1 suppresses ferroptosis by enhancing FSP1 N-myristoylation, whereas the long non-coding RNA CACNA1G-AS1 promotes tumour progression through FTH1 upregulation and subsequent inhibition of ferroptosis [106]. Transcriptomic analyses have further expanded the therapeutic landscape, with Zhang et al. identifying 15 ferroptosis-related mRNAs as potential therapeutic targets for ovarian cancer. A variety of ferroptosis-inducing agents have also demonstrated promising anti-tumour activity. Lidocaine enhances ferroptosis by upregulating miR-382-5p and suppressing SLC7A11, while the combination of the mixed-lineage leukaemia antagonist MI-463 and auranofin synergistically induces ferroptotic cell death. Similarly, superparamagnetic iron oxide nanoparticles (SPIONs), artemisinin, and the combination of MAP30, erastin, and cisplatin (cisplatin) effectively inhibit ovarian cancer growth by promoting reactive oxygen species accumulation and ferroptosis. In addition, promyelocytic leukaemia protein (PML) has been shown to enhance chemosensitivity through ferroptosis induction [10, 54, 79, 107, 108].

Resistance to ferroptosis is also regulated by several metabolic pathways in ovarian cancer. Liu et al. demonstrated that activation of the NRF2/CBS signalling pathway enhances transsulfuration activity, thereby protecting tumour cells from erastin-induced ferroptosis. Likewise, Carbone et al. reported that stearoyl-CoA desaturase 1 (SCD1) deficiency suppresses tumour growth by promoting ferroptosis, highlighting metabolic reprogramming as a promising therapeutic strategy for overcoming ferroptosis resistance in ovarian cancer [109].

### Hepatocellular carcinoma

In hepatocellular carcinoma (HCC), the clearest clinical signal to date comes from gene signatures: ferroptosis-related panels stratify patients by survival risk and predict immune microenvironment composition and treatment response. These prognostic models not only stratify patients according to survival risk but also facilitate the identification of individuals who are more likely to benefit from immunotherapy and targeted therapies. For example, Tang et al. developed a ferroptosis-based predictive model that accurately estimated the infiltration of immune cell populations, including macrophages, neutrophils, and memory B cells, thereby providing insights into the immune landscape of HCC [110]. Similarly, Liu et al. demonstrated that elevated expression of ferroptosis suppressors, together with increased infiltration of immunosuppressive cells and evidence of bone marrow suppression, was strongly associated with unfavourable clinical outcomes [111]. Collectively, these findings indicate that ferroptosis-related molecular signatures possess considerable prognostic and predictive value, supporting their potential application in patient stratification and personalised therapeutic decision-making for HCC [111, 112].

Therapeutically, several ferroptosis-inducing compounds show activity in preclinical HCC models. The kinase inhibitor donafenib acts synergistically with GSK-J4 to induce ferroptosis through HMOX1 regulation, resulting in significant suppression of HCC growth [113, 114]. Similarly, dihydroartemisinin (DHA) promotes the formation of the PEBP1–ALOX15 complex, thereby enhancing lipid peroxidation and ferroptotic cell death. Other ferroptosis-promoting agents, including atractylodin, increase intracellular reactive oxygen species by upregulating ACSL4 and TFRC while suppressing FTL1 and GPX4 expressions. Likewise, low-density lipoprotein docosahexaenoic acid nanoparticles induce ferroptosis by elevating lipid hydroperoxide accumulation and inhibiting GPX4 activity in HCC cells [115, 116]. These findings highlight ferroptosis induction as a promising therapeutic strategy for HCC. Ferroptosis has also been closely linked to sorafenib responsiveness, one of the major challenges in HCC management. Suppression of the p62/NRF2 signalling pathway sensitises HCC cells to erastin- and sorafenib-induced ferroptosis, thereby enhancing the antitumour efficacy of sorafenib. Consistently, Yao et al. demonstrated that reduced LCN2 expression promotes ferroptosis and significantly potentiates the therapeutic effects of sorafenib, suggesting that targeting ferroptosis may represent an effective approach for overcoming sorafenib resistance in HCC [117].

Several molecular regulators have recently been identified that influence ferroptosis sensitivity and therapeutic resistance in hepatocellular carcinoma (HCC). ABCC5 suppresses sorafenib-induced ferroptosis by stabilising SLC7A11 and increasing intracellular glutathione (GSH) levels, whereas activation of the YAP/TAZ signalling pathway similarly promotes sorafenib resistance through transcriptional upregulation of SLC7A11 [118]. Conversely, tiliroside enhances ferroptosis by inhibiting TBK1, thereby restoring the sensitivity of HCC cells to sorafenib. In addition, SOCS2 promotes ferroptosis through ubiquitination-mediated degradation of SLC7A11, resulting in increased radiosensitivity, while suppression of PGAM1 induces ferroptosis and enhances the efficacy of immunotherapy. The mitochondrial protein TSPO, however, facilitates HCC progression by activating the NRF2-dependent antioxidant defence pathway, thereby limiting ferroptotic cell death [119]. Metabolic regulation also contributes to ferroptosis susceptibility, as the HCAR1/MCT1 axis promotes ferroptosis by reducing lactate uptake, activating AMPK, and suppressing SCD1 expression, highlighting lactate metabolism as a potential therapeutic target. Furthermore, maintenance of iron homeostasis by recombinant ferritin heavy chain (FTH) protein reduces mitochondrial reactive oxygen species accumulation, limits lipid peroxide formation, preserves mitochondrial function, and consequently suppresses ferroptosis in HCC cells [116].

### Lung cancer

Two distinct lines of work converge in lung cancer, one prognostic and one pharmacological. Ferroptosis-related gene signatures predict patient prognosis, characterise the tumour immune microenvironment, and identify individuals most likely to benefit from immunotherapy. For example, Teng et al. established a prognostic model integrating ferroptosis- and immune-related genes, whereas Zhang et al. demonstrated that the ferroptosis score closely correlates with tumour immune phenotypes. A high ferroptosis score was associated with an immunosuppressive microenvironment, while a low ferroptosis score reflected enhanced immune activation and improved responsiveness to anti-PD-1/PD-L1 immunotherapy, highlighting the clinical utility of ferroptosis-based stratification in lung cancer management [120]. Beyond prognostic applications, numerous ferroptosis-inducing agents have demonstrated promising anti-tumour activity in lung cancer. β-Elemene enhances erlotinib sensitivity by promoting ferroptosis through lncRNA H19 upregulation, whereas the natural compound manoalide suppresses the NRF2–SLC7A11 signalling axis to induce ferroptotic cell death. Similarly, bufotalin promotes GPX4 ubiquitination, and zinc enhances intracellular iron, malondialdehyde (MDA), and reactive oxygen species (ROS) accumulation through inhibition of circFOXP1, collectively suppressing tumour growth [121, 122]. Furthermore, combined treatment with erastin and celastrol markedly enhances ferroptosis by promoting mitochondrial disruption, autophagy, oxidative stress, and interferon-γ (IFN-γ) release from cytotoxic T lymphocytes, thereby reinforcing anti-tumour immune responses [123].

Regulation converges particularly on System Xc⁻: SOX2 transcriptionally activates SLC7A11, thereby suppressing ferroptosis, whereas miR-27a-3p promotes ferroptotic cell death by directly targeting the 3′-UTR of SLC7A11. In contrast, RBMS1 binds both the 3′- and 5′-UTRs of SLC7A11 to enhance its translation and confer ferroptosis resistance. Metabolic regulation also contributes to ferroptosis susceptibility, as exogenous glutamate sensitises lung cancer cells to ferroptosis by suppressing YAP signalling in an ADCY10-dependent manner following System Xc⁻ inhibition, while the m6A reader YTHDC2 promotes ferroptosis through inhibition of SLC3A2 [106].

Additional regulatory pathways further influence ferroptosis in lung cancer. MIB1 promotes ferroptosis by facilitating lysosomal degradation of NRF2, whereas USP11 stabilises NRF2 through deubiquitination and consequently inhibits ferroptotic cell death [61]. Acetaminophen enhances erastin sensitivity by suppressing NRF2 transcription and reducing HMOX1 expression, while activation of the GSK3β–β-TrCP axis induces ferroptosis through inhibition of the AMPK/mTOR–NRF2 pathway. Moreover, USP35 attenuates ferroptosis by preventing ferroportin (FPN) degradation, whereas inhibition of AKR1C1 effectively induces ferroptosis and suppresses lung cancer progression [124].

### Prostate Cancer

Erastin and RSL3 both suppress prostate cancer cell proliferation and metastasis by promoting intracellular reactive oxygen species (ROS) accumulation and lipid peroxidation. Notably, RSL3 enhances the antitumour efficacy of cisplatin (cisplatin) by disrupting glycolytic metabolism and potentiating cell-cycle arrest. Furthermore, elevated intracellular iron levels have been shown to improve the response to antiandrogen therapy through enhanced lipid peroxidation and ferroptosis induction, highlighting the therapeutic potential of targeting iron metabolism in prostate cancer [125].

Ferroptosis has also emerged as an important mechanism underlying therapeutic resistance, particularly in advanced prostate cancer. A ROS-amplifying multimodal nanoplatform has demonstrated the ability to overcome multidrug resistance, while Ingram et al. reported that ferroptosis is closely associated with docetaxel resistance using nanocancer cell models [126]. Consistently, erastin restores docetaxel sensitivity by suppressing the transcriptional activity of androgen receptors and their splice variants. In castration-resistant prostate cancer (CRPC), the anthelmintic flubendazole induces ferroptosis by activating p53, repressing SLC7A11 transcription, and reducing GPX4 expression [127]. Traditional Chinese medicines have also shown therapeutic potential, with Qiling Decoction promoting ferroptosis through FSP1 inhibition, whereas luteolin enhances TFEB-dependent ferritinophagy, thereby increasing intracellular iron availability.

Among other regulators, SGK2 promotes tumour metastasis by increasing GPX4 expression and suppressing ferroptosis, whereas supraphysiological testosterone concentrations induce ferroptosis through activation of ferritinophagy [128]. The interfering peptide B8R enhances ferroptosis by activating p53, increasing p38 phosphorylation, and promoting intracellular iron and ROS accumulation. In contrast, lncRNA OIP5-AS1 confers ferroptosis resistance through the miR-128-3p/SLC7A11 signalling pathway. Metabolic regulators also contribute to ferroptosis, as 2,4-dienoyl-CoA reductase (DECR) promotes mitochondrial oxidative stress and lipid peroxidation and has emerged as a potential biomarker for castration-resistant prostate cancer. Conversely, the ATPase VCP suppresses ferroptosis by preserving mitochondrial function and limiting glutathione (GSH) consumption [129, 130].

### Breast cancer

Nanomedicine has dominated ferroptosis research in breast cancer. Oxygen self-generating nanoreactors enhance immunotherapy through GPX4 depletion, whereas metal–polyphenol network-coated Prussian blue nanoparticles and azobenzene combretastatin A4 nanospheres suppress tumour growth by promoting ferroptotic cell death. Likewise, a heparinase-responsive nanoparticle delivery system inhibits breast cancer metastasis by increasing intracellular reactive oxygen species (ROS) accumulation and enhancing ferroptosis [131, 132]. In addition, lapatinib has been shown to induce ferroptosis across multiple breast cancer models, suggesting that ferroptosis induction may represent an effective strategy for overcoming therapeutic resistance [133].

Ferroptosis also provides opportunities for drug repurposing and the development of novel therapeutic combinations. Metformin and simvastatin suppress tumour growth by reducing the expression of SLC7A11 and GPX4, respectively, thereby promoting ferroptosis. Several naturally occurring compounds have demonstrated similar activity, including isoliquiritigenin, which alleviates doxorubicin resistance through inhibition of the NF-κB signalling pathway, as well as curcumin, DMOCPTL, and glycyrrhetinic acid, which induce ferroptosis by modulating redox-associated genes, targeting GPX4, and activating NADPH oxidase and inducible nitric oxide synthase (iNOS) [86, 134]. Furthermore, Prominin2 has recently been recognised as a key regulator of ferroptosis resistance, highlighting the importance of metabolic adaptation in breast cancer progression [135].

Several signalling molecules further regulate ferroptosis sensitivity in breast cancer. TYRO3 promotes resistance to immunotherapy by suppressing ferroptosis and reducing the M1/M2 macrophage ratio, whereas DDR2 enhances ferroptosis susceptibility through activation of the Hippo signalling pathway. In contrast, hepatic leukaemia factor (HLF) promotes ferroptosis resistance by transcriptionally activating GGT1, further illustrating the complex regulatory network governing ferroptosis in breast cancer [39].

### Colorectal cancer

Much of the colorectal cancer (CRC) literature centres on natural products. Osthole, talaroconvolutin A, and 2-imino-6-methoxy-2 H-chromene-3-carbothioamide promote ferroptotic cell death by suppressing SLC7A11 expression or inhibiting the AMPK/Akt signalling pathway [136]. Notably, Xia et al. identified talaroconvolutin A as a novel ferroptosis inducer exhibiting greater efficacy than erastin. Combination approaches have also shown encouraging therapeutic potential, with dihydroartemisinin (DHA) and pyropheophorbide iron synergistically inducing ferroptosis and enhancing the responsiveness of colorectal tumours to anti-PD-L1 immunotherapy [10]. Likewise, elesclomol triggers copper-dependent ferroptosis through degradation of the copper transporter ATP7A, whereas iron oxyhydroxide and zinc oxide nanospheres inhibit tumour growth by promoting ferroptotic cell death. Furthermore, cetuximab enhances RSL3-induced ferroptosis through inhibition of the p38/NRF2/HMOX1 signalling pathway, while both β-elemene and vitamin C further sensitise colorectal cancer cells to cetuximab by augmenting ferroptosis [137].

Recent studies have also identified several molecular regulators that influence ferroptosis sensitivity and therapeutic resistance in colorectal cancer. CYP1B1 promotes resistance to PD-1 blockade by facilitating ACSL4 degradation, thereby suppressing ferroptosis. In contrast, both OTUD1 and HIF-2α enhance intracellular reactive oxygen species (ROS) accumulation and promote ferroptotic cell death. Conversely, lipocalin-2 (LCN2) facilitates tumour progression by activating GPX4 and reducing intracellular iron availability, highlighting the importance of iron metabolism and antioxidant defence in regulating ferroptosis susceptibility in colorectal cancer.

### Gastric cancer

Gastric cancer is notable for the prominence of non-coding RNA regulation. Ferroptosis-related lncRNA signatures reflect tumour biology, immune microenvironment, genomic stability, and treatment responsiveness, thereby facilitating the prediction of outcomes and the selection of chemotherapeutic agents, including methotrexate, paclitaxel, and gemcitabine, as well as immunotherapeutic strategies [138]. A variety of pharmacological agents have also demonstrated potent anti-tumour activity by inducing ferroptosis. Polyphyllin-I suppresses gastric cancer growth through downregulation of NRF3 and FTH1, whereas levobupivacaine promotes ferroptosis by increasing miR-489-3p expression and inhibiting SLC7A11. Similarly, apatinib and tanshinone IIA trigger ferroptosis by depleting glutathione (GSH) and enhancing lipid peroxidation, while oridonin A and extracts of Andrographis paniculata further inhibit tumour progression through ferroptosis induction [139].

Ferroptosis is also closely associated with therapeutic resistance in gastric cancer. Inhibition of SIRT6 promotes ferroptosis by suppressing GPX4 activity and disrupting the KEAP1/NRF2 signalling pathway, thereby enhancing sensitivity to sorafenib. Likewise, ATF3 overcomes cisplatin (cisplatin) resistance by inhibiting the NRF2/KEAP1/System Xc⁻ axis and promoting ferroptotic cell death. In contrast, miR-522, secreted by tumour-associated fibroblasts, activates the hnRNPA1/USP7 signalling pathway, suppresses ALOX15, reduces reactive oxygen species accumulation, and ultimately confers chemoresistance through inhibition of ferroptosis [140].

CTS1 promotes tumour metastasis by stabilising GPX4 and suppressing ferroptosis, whereas miR-375 directly targets SLC7A11 to induce ferroptotic cell death [141]. Moreover, perilipin-2 and exosomal lncFERO inhibit ferroptosis by downregulating ACSL3, ALOX15, and PRDM11, while upregulating SCD1. Conversely, CPEB1 promotes ferroptosis through repression of TWIST1 and activation of the ATF4/CHAC1 pathway, whereas loss of CDO1 expression attenuates ferroptosis and supports gastric cancer progression [10].

### Pancreatic cancer

A study showed that GPX4 deletion promoted the occurrence of pancreatic cancer in mice and this action was driven by KRAS^G12D^ and reversed by liproxstatin-1 [1]. Dai et al. demonstrated that ferroptotic cells secreted the KRAS^G12D^ protein and facilitated macrophage differentiation into the M2 subtype. This study suggested that ferroptosis contributed to cancer cell development by inhibiting immune cell responses [142]. These results indicated that ferroptosis contributed to the progression of pancreatic cancer. However, the absence of SLC7A11 or Cys suppressed the development of pancreatic cancer, implying that ferroptosis negatively affects cancer cells. Moreover, Bhutia et al. showed that mutant TP53 might be a checkpoint for ferroptosis-mediated tumour formation and suppression. Therefore, elucidating the function of TP53 in ferroptosis is essential for pancreatic cancer treatment [143].

In addition, SLC38A5 could be used as a sensitising target in pancreatic cancer by regulating ferroptosis. GOT1 and NUPR1 knockdown contributed to ferroptosis in pancreatic cancer cells. Tomatidine, wogonin and dithiocarbazate–copper complexes exert anticancer effects by inducing ferroptosis in pancreatic cancer. DHA increased the sensitivity of pancreatic cancer cells to CDDP by increasing free iron levels and triggering ferroptosis [144].

### Renal cancer

Renal cancer occupies a distinctive position, since clear-cell tumours are intrinsically ferroptosis-competent (see above). Against this background, PDIA4 promotes ferroptosis resistance through activation of the ATF4/SLC7A11 signalling pathway, thereby enhancing antioxidant defence in renal cancer cells. Conversely, renal-clearable ultrasmall single-crystal iron (Fe) nanoparticles have emerged as a novel immunotherapeutic delivery platform capable of inducing ferroptosis and improving antitumour efficacy. Epigenetic regulation also plays a pivotal role in ferroptosis, as loss of the histone demethylase KDM5C increases intracellular G6PD, NADPH, and glutathione (GSH) levels, thereby suppressing ferroptosis, whereas deletion of the histone methyltransferase SUV39H1 promotes ferroptotic cell death. In addition, the transcription factor KLF2 induces ferroptosis by suppressing GPX4 expression, while activation of the Hippo effector TAZ enhances NOX4 expression to increase oxidative stress and ferroptosis. Furthermore, fumarate hydratase promotes C93 succination of GPX4, impairing its antioxidant activity and thereby sensitising renal cancer cells to ferroptosis [145, 146].

### Others

Ferroptosis also contributes to other malignancies. In glioma, neutrophils induce tumour cell ferroptosis by transferring myeloperoxidase into cancer cells, while in neuroblastoma MYCN increases iron sensitivity and suppresses the glutathione defence system [147]. Erastin and its derivatives imidazole ketone erastin and piperazine erastin, inhibit growth of melanoma, cervical cancer, diffuse large B-cell lymphoma and fibrosarcoma [139].

## Ferroptosis in epithelial–mesenchymal transition, invasion and metastasis

Ferroptosis plays a pivotal role in cancer progression by regulating epithelial–mesenchymal transition (EMT), tumour invasion, migration, and metastatic dissemination [15]. Accumulating evidence indicates a bidirectional relationship between ferroptosis and EMT, whereby each process influences the other. On one hand, ferroptosis suppresses EMT and limits tumour progression. Increased reactive oxygen species (ROS) accumulation and ferritinophagy inhibit EMT by enhancing p53 expression while suppressing the AKT/mTOR signalling pathway, whereas the ferroptosis inhibitor ferrostatin-1 reverses these effects [148]. Likewise, activation of the Keap1/NRF2/HO-1 signalling pathway together with ferritinophagy promotes ferroptosis and suppresses EMT in gastric cancer cells. Conversely, acquisition of the mesenchymal phenotype increases susceptibility to ferroptosis. Transforming growth factor-β1 (TGF-β1)-induced EMT sensitises tumour cells to ferroptosis by reducing SLC7A11 expression and glutathione (GSH) levels while increasing malondialdehyde (MDA) and ROS accumulation. In addition, discoidin domain receptor tyrosine kinase 2 (DDR2) activates the YAP/TAZ signalling pathway during the early stages of EMT, further enhancing ferroptosis sensitivity in breast cancer [149].

Beyond regulating EMT, ferroptosis also directly influences the invasive and migratory behaviour of tumour cells through alterations in redox homeostasis and lipid metabolism. Elevated GPX4 expression is associated with enhanced invasion and migration in renal cancer, whereas GSH depletion diminishes migratory capacity, highlighting the importance of antioxidant defence in metastatic progression [20]. Similarly, SLC7A11 promotes tumour invasion by sustaining GPX4-mediated ferroptosis resistance and maintaining cellular redox balance. However, the relationship is not universally suppressive. ACSL4, a central regulator of ferroptosis, has been linked to a more invasive phenotype, and accumulation of lipid peroxidation products, including PUFA-CoA and PUFA-containing phospholipids, may promote invasion and migration in selected tumour models. These apparently opposing findings suggest that the impact of ferroptosis on tumour invasion and metastasis is highly context-dependent, varying with tumour type, metabolic state, and microenvironmental conditions. Consequently, therapeutic modulation of ferroptosis requires careful consideration of tumour-specific biology to maximise its anti-metastatic potential while minimising unintended pro-invasive effects [20].

## Resolving the ferroptosis paradox: determinants of tumour-suppressive versus tumour-promoting outcomes

Ferroptosis is widely regarded as a tumour-suppressive mechanism, yet a substantial body of evidence indicates that it can also promote inflammation, immune suppression, metastasis and tumour evolution. This apparent paradox is among the most consequential unresolved issues in the field, since it determines whether ferroptosis induction is therapeutically advantageous in a given setting. The contradiction is more apparent than real: the biochemistry of ferroptosis is invariant, but its consequences depend on which cell undergoes ferroptosis, at what stage of disease, what is released upon death, and whether the resulting corpses are efficiently cleared.

### Cellular identity is the principal determinant

Ferroptosis of tumour cells reduces tumour burden, whereas ferroptosis of immune effector cells impairs anti-tumour immunity. Tumour-derived cholesterol drives CD36 overexpression in CD8⁺ T cells, increasing lipid peroxidation and ferroptosis while compromising cytotoxicity; cancer-associated fibroblasts expand the labile iron pool in natural killer cells and induce NCOA4-dependent ferritinophagy, impairing their function; and ferroptotic polymorphonuclear myeloid-derived suppressor cells release oxygenated lipids that suppress T-cell activity. Non-selective ferroptosis induction may therefore simultaneously kill tumour cells and disarm the immune compartment required for durable response, which provides a rationale for tumour-restricted approaches such as selective GPX4 degradation [150].

### Disease stage inverts the outcome

In pancreatic cancer, high-iron diets or pancreatic Gpx4 depletion accelerate Kras-driven tumorigenesis, and administration of the ferroptosis inhibitor liproxstatin-1 suppresses tumour development in this pre-malignant context [151]. Once carcinoma is established, by contrast, ferroptosis induction inhibits growth. An analogous stage-dependence is observed in the liver, where ferroptosis contributes to progression from chronic injury to hepatocellular carcinoma but restrains established disease. Ferroptosis induction may therefore be contraindicated in settings of chronic tissue injury and pre-neoplasia, even where it is beneficial against established tumours.

### The composition of released material determines the immune consequence

Oxidised phospholipids such as SAPE-OOH act as “eat-me” signals promoting macrophage clearance of ferroptotic cells, whereas release of 8-hydroxyguanosine activates STING1-dependent macrophage infiltration and M2 polarisation, supporting tumorigenesis [151], and autophagy-dependent release of KRAS^G12D^ protein drives M2 polarisation through AGER signalling. Ferroptosis is thus not uniformly immunogenic; whether it is immunostimulatory or immunosuppressive depends on the DAMP profile generated.

### Clearance capacity modulates all of the above

Excessive phospholipid peroxidation within tumour-associated macrophages impairs their phagocytic function and reduces ferroptotic cell clearance, favouring persistent oxidative and inflammatory signalling rather than resolution. Chronic, low-level ferroptosis with impaired clearance may consequently generate a mutagenic and immunosuppressive microenvironment promoting tumour evolution, whereas acute, efficiently cleared ferroptosis is more likely to be therapeutically beneficial.

Taken together, these determinants suggest that the therapeutic question is not whether to induce ferroptosis but where, when, in which cell population, and with what kinetics. This reframing has direct implications for trial design, favouring tumour-selective delivery, established rather than pre-neoplastic disease, and combination with agents that preserve or restore immune effector function.

## Ferroptosis in the tumour microenvironment

The tumour microenvironment (TME) comprises multiple components, encompassing not only tumour cells but also stromal and immune cells. Each cell type in the TME can be further characterised by multiple markers to exhibit a range of phenotypes. These components are organised in spatially structured arrangements, showing microenvironmental niches, nutrient gradients, and cell-cell interaction phenotypes. Multiple non-tumour cell types in the TMEm, such as cancer-associated fibroblasts (CAFs) and tumour-associated macrophages (TAMs), drive a chronic inflammatory, immunosuppressive, and pro-angiogenic intratumoural environment that supports tumour growth and metastasis. Ferroptotic cell-released metabolites or damage-associated molecular patterns in the TME can induce an immune response of tumour-infiltrating immune cells, suggesting ferroptosis as an immunogenic cell death [1, 76].

### Ferroptosis-derived signals as mediators of intercellular communication

Ferroptotic tumour cells are not passive casualties within the tumour microenvironment (TME); they release a structurally diverse set of mediators that actively reshape surrounding immune and stromal populations. Three broad classes have been characterised.

Oxidised phospholipids constitute the most specific class, since their generation is intrinsic to the ferroptotic mechanism itself. These species function as signalling molecules rather than merely as damage products: 1-stearoyl-2-15-HpETE-*sn*-glycero-3-phosphatidylethanolamine (SAPE-OOH) is externalised on the surface of ferroptotic cells and acts as an “eat-me” signal recognised by TLR2 on macrophages, thereby directing their clearance. Oxidised phosphatidylethanolamine species exert signalling activity beyond phagocyte recognition, and analogous oxidised phospholipid-driven modulation of inflammatory and immune responses has been described in non-malignant immune-mediated disease, indicating that these mediators represent a general mechanism of ferroptosis-associated intercellular communication rather than a tumour-restricted phenomenon [152].

Reactive aldehydes are generated as secondary decomposition products of lipid peroxidation. Malondialdehyde and 4-hydroxynonenal form protein and DNA adducts, propagate oxidative stress into neighbouring cells, and amplify inflammatory signalling, extending the biochemical consequences of ferroptosis beyond the dying cell. Because these species are diffusible and relatively stable, they may act at a distance from the site of ferroptotic death.

Damage-associated molecular patterns and lipid mediators constitute the third class. Ferroptotic cells release high mobility group box 1 (HMGB1), 8-hydroxyguanosine and prostaglandin E2, which together regulate macrophage activation and inflammatory tone. Autophagy-dependent ferroptosis additionally releases KRAS^G12D^ protein, which is taken up by macrophages via AGER and drives M2 polarisation through fatty acid oxidation. Ferroptotic polymorphonuclear myeloid-derived suppressor cells release oxygenated lipids that directly suppress T-cell function, demonstrating that this signalling operates between immune populations as well as from tumour to stroma.

The functional consequence of this output is therefore not fixed but depends on its composition, on the capacity of tumour-associated macrophages to clear ferroptotic corpses, and on the disease context in which it occurs, as discussed above.

### Ferroptosis and CAFs in the TME

Cancer-associated fibroblasts (CAFs) are among the most abundant stromal components of the tumour microenvironment (TME) and play fundamental roles in tumour progression. Originating from mesenchymal stromal cells, CAFs exhibit spindle-shaped morphology and distinct phenotypic characteristics compared with quiescent fibroblasts in normal tissues. Although historically regarded as a homogeneous population of extracellular matrix-producing cells, recent single-cell RNA sequencing (scRNA-seq) studies have revealed substantial phenotypic and functional heterogeneity, with specific CAF subpopulations exerting diverse effects on tumour progression and therapeutic response [153]. Emerging evidence indicates that CAFs also regulate ferroptosis susceptibility in tumour cells. In nasopharyngeal carcinoma, CAF-derived fibroblast growth factor 5 (FGF5) activates fibroblast growth factor receptor 2 (FGFR2) signalling in tumour cells, thereby suppressing cisplatin-induced ferroptosis. Likewise, in pancreatic ductal adenocarcinoma, CAFs enhance extracellular cysteine availability through the TGF-β/SMAD3/ATF4 signalling pathway, promoting glutathione (GSH) synthesis and conferring resistance to ferroptosis [154, 155].

The interaction between tumour cells and CAFs is reciprocal, with ferroptosis in tumour cells also shaping CAF recruitment and function. In gastrointestinal cancers, overexpression of anoctamin-1 (ANO1) suppresses ferroptosis through activation of the PI3K–Akt signalling pathway while simultaneously increasing transforming growth factor-β (TGF-β) secretion. This promotes CAF recruitment, remodels the tumour microenvironment, and contributes to resistance to immunotherapy, highlighting the dynamic interplay between ferroptosis and stromal remodelling [156]. Extracellular vesicles (EVs), including exosomes and microvesicles, have also emerged as important mediators of ferroptosis-related communication within the TME. By transporting proteins, lipids, and nucleic acids between tumour cells and stromal components, including CAFs and macrophages, EVs coordinate intercellular signalling that influences ferroptosis sensitivity, tumour progression, immune regulation, and therapeutic responses, thereby representing an additional layer of ferroptosis-mediated tumour–stroma crosstalk [157].

### Ferroptosis and Myeloid Cells in the TME

Myeloid cells constitute the largest population of innate immune cells within the tumour microenvironment (TME) and exhibit both immunostimulatory and immunosuppressive functions. This heterogeneous compartment is composed primarily of macrophages, neutrophils, and dendritic cells (DCs), all of which contribute to tumour progression and therapeutic responses. Among these, tumour-associated macrophages (TAMs) are key regulators of the immunosuppressive TME. TAMs are broadly classified into M1-like and M2-like phenotypes, with M1 macrophages promoting anti-tumour immunity through the production of pro-inflammatory and angiostatic mediators, whereas M2 macrophages facilitate tumour growth by secreting tissue-remodelling and pro-angiogenic factors that support tumour progression [158].

Accumulating evidence indicates that ferroptosis is closely linked to macrophage biology and function. The first evidence of macrophage ferroptosis was reported in 2017, when Wang et al. demonstrated that iron overload induces SLC7A11-dependent ferroptosis in murine hepatocytes and macrophages [159]. Beyond ferroptosis induction, macrophages are essential for the clearance of ferroptotic tumour cells during anticancer therapy. Luo et al. identified 1-stearoyl-2-15-HpETE-sn-glycero-3-phosphatidylethanolamine (SAPE-OOH) as a critical oxidised phospholipid “eat-me” signal that promotes macrophage-mediated phagocytosis of ferroptotic tumour cells. However, excessive phospholipid peroxidation within TAMs impairs their phagocytic capacity, thereby reducing ferroptotic cell clearance and contributing to resistance against ferroptosis-based therapies. Mechanistically, SAPE-OOH promotes TLR2 ubiquitin-dependent degradation, thereby disrupting macrophage recognition of ferroptotic cells [61, 160].

Ferroptosis also influences macrophage polarisation and intercellular communication within the TME. Tumour-derived exosomes carrying oncogenic KRAS are internalised by macrophages, inducing metabolic reprogramming that favours M2 polarisation and tumour progression [161, 162]. In addition, ferroptotic tumour cells actively remodel the innate immune microenvironment through the release of damage-associated molecular patterns (DAMPs) and lipid mediators, including high mobility group box 1 (HMGB1), 8-hydroxyguanosine (8-OHG), and prostaglandin E2 (PGE2). These signals regulate macrophage activation and inflammatory responses, highlighting the complex bidirectional crosstalk between ferroptosis and macrophages within the tumour microenvironment [163].

### Neutrophils

Neutrophils are increasingly recognised as important regulators of ferroptosis within the tumour microenvironment (TME), exerting both tumour-promoting and tumour-suppressive functions. The association between neutrophils and oxidative stress was first highlighted by Knaapen et al. in 1999, who demonstrated that neutrophil-derived reactive oxygen species (ROS) induce oxidative DNA damage in epithelial cells, providing early evidence linking neutrophils to ferroptosis-related mechanisms. More recently, single-cell RNA sequencing (scRNA-seq) has revealed marked functional heterogeneity among tumour-infiltrating neutrophils. In hepatoblastoma, CXCR4⁺ neutrophils display increased susceptibility to ferroptosis together with an immunosuppressive phenotype, whereas Acod1⁺ tumour-associated neutrophils in breast cancer exhibit resistance to ferroptosis and promote metastatic progression through Acod1-dependent immunometabolic reprogramming. These findings demonstrate that the influence of ferroptosis on neutrophil function is highly context dependent and varies across tumour types [164, 165].

Beyond tumour progression, neutrophil-mediated ferroptosis also contributes to cancer-associated cachexia, a syndrome responsible for a substantial proportion of cancer-related mortality. In lung cancer, infiltrating neutrophils secrete lipocalin-2 (LCN2), which induces ferroptosis and accelerates adipose and skeletal muscle wasting. Neutrophils also promote tumour necrosis, a marker of poor prognosis in advanced malignancies, by transferring myeloperoxidase (MPO)-containing granules that trigger ferroptosis in glioblastoma cells. In addition, immunosuppressive neutrophils, including polymorphonuclear myeloid-derived suppressor cells (PMN-MDSCs) and tumour-associated neutrophils (TANs), modulate antitumour immunity through ferroptosis. Ferroptotic PMN-MDSCs release oxygenated lipids that suppress T-cell activity, thereby reinforcing an immunosuppressive tumour microenvironment, while ferroptosis may further facilitate tumour progression by promoting MDSC recruitment. Collectively, these findings identify neutrophil ferroptosis as a promising therapeutic target for modulating tumour immunity and improving cancer treatment outcomes [8, 165].

### T-lymphocytes

T lymphocytes are central mediators of adaptive immunity and play indispensable roles in tumour immune surveillance and immunotherapy. Derived from bone marrow progenitors and matured within the thymus, T cells are broadly classified into CD8⁺ cytotoxic and CD4⁺ helper T-cell subsets. Among these, the interplay between CD8⁺ T cells and ferroptosis has received considerable attention. Although CD8⁺ T cells were traditionally regarded as uniformly cytotoxic cells that eliminate malignant cells through the release of interferon-γ (IFN-γ) and cytolytic granules, recent studies have revealed substantial functional heterogeneity within the tumour microenvironment (TME). Distinct exhausted and immunosuppressive CD8⁺ T-cell populations exhibit impaired cytotoxic activity and contribute to tumour immune escape [166].

The TME profoundly influences the ferroptosis susceptibility of CD8⁺ T cells. Tumour-derived cholesterol promotes CD36 overexpression, leading to increased fatty acid uptake, lipid peroxidation, and ferroptosis, thereby compromising T-cell-mediated cytotoxicity. In contrast, Tc9 cells display intrinsic resistance to ferroptosis through IL-9/STAT3-mediated enhancement of fatty acid oxidation and suppression of lipid peroxidation. Additional protective mechanisms include DEPDC5-mediated inhibition of mTORC1-dependent purine catabolism, while blockade of the PGE2–EP2/EP4 signalling axis alleviates oxidative stress-induced ferroptosis and enhances the efficacy of adoptive T-cell therapy [167]. The interaction between ferroptosis and T cells is reciprocal, as activated CD8⁺ T cells actively induce ferroptosis in tumour cells. Secretion of IFN-γ suppresses SLC3A2 and SLC7A11, restricting cystine uptake, promoting lipid peroxidation, and sensitising tumour cells to ferroptosis. IFN-γ further enhances ACSL4 expression, thereby improving responses to radiotherapy and immune checkpoint blockade. Similarly, restoration of MAT1A expression augments CD8⁺ T-cell function while promoting ferroptosis in hepatocellular carcinoma, highlighting the synergistic relationship between adaptive immunity and ferroptotic cell death [168].

In contrast, regulatory T cells (Tregs) suppress antitumour immunity and maintain resistance to ferroptosis through GPX4-dependent antioxidant defence. Genetic deletion of GPX4 induces ferroptosis in Tregs, resulting in reduced tumour growth and enhanced antitumour immune responses. Likewise, LINC00942 facilitates immune evasion by stabilising SLC7A11 mRNA, thereby suppressing ferroptosis and reinforcing Treg-mediated immunosuppression [169].

### Natural killer (NK) cells in the TME

Natural killer (NK) cells are specialised innate lymphoid cells that provide rapid immune surveillance against malignant and virus-infected cells through their intrinsic cytotoxic activity. Despite their potent antitumour functions, NK cells within the tumour microenvironment (TME) frequently acquire a dysfunctional phenotype characterised by impaired cytotoxicity, making restoration of NK-cell activity an attractive strategy for cancer immunotherapy [170]. Recent evidence indicates that ferroptosis is an important mechanism underlying NK-cell dysfunction. Tumour cells and stromal components within the TME can promote NK-cell ferroptosis through aryl hydrocarbon receptor-independent mechanisms, thereby limiting their antitumour activity. Among these stromal regulators, cancer-associated fibroblasts (CAFs) play a particularly important role. Yao et al. demonstrated that CAFs increase iron availability within the gastric cancer microenvironment, resulting in expansion of the labile iron pool in NK cells and subsequent induction of ferroptosis. In addition, CAF-derived follistatin-like protein 1 (FSTL1) upregulates NCOA4, promoting NCOA4-dependent ferritinophagy and further sensitising NK cells to ferroptotic cell death. Collectively, these findings suggest that targeting ferroptosis in NK cells may restore their cytotoxic function and improve the efficacy of NK cell-based immunotherapies [171].

## Ferroptosis-related therapeutic strategies in the tumour microenvironment

### Classification of ferroptosis inducers

Ferroptosis-inducing agents (FINs) are conventionally grouped into four classes according to the node of the ferroptosis network at which they act, a framework that clarifies both their expected mechanism of resistance and their rational combination partners [172].

Class I FINs deplete glutathione by inhibiting cystine import through System Xc⁻. This class includes erastin and its analogues, imidazole ketone erastin and piperazine erastin, sulfasalazine, sorafenib, and glutamate. Because glutathione depletion leaves GPX4 protein intact, Class I agents are ineffective where transsulfuration flux or alternative cysteine sources are available.

Class II FINs inhibit GPX4 directly, typically through covalent modification of its catalytic selenocysteine. RSL3, ML162 and altretamine act in this manner. Unlike Class I agents, they bypass cysteine availability entirely, but their activity is opposed by GPX4-independent arms, particularly FSP1.

Class III FINs deplete both GPX4 protein and coenzyme Q₁₀ by acting on the mevalonate pathway, exemplified by FIN56, which induces GPX4 degradation while concurrently reducing CoQ₁₀ availability [31].

Class IV FINs initiate lipid peroxidation directly through iron overload or oxidation of labile iron, rather than by disabling antioxidant defence. FINO2, iron-based nanomaterials, and iron supplementation act in this way, and this class has attracted particular attention in iron-addicted tumours such as epithelial ovarian cancer, in which increased iron uptake and reduced export generate a pre-existing vulnerability [173, 174].

This classification maps directly onto the defence network architecture described earlier: Class I and II agents target a single arm and are therefore susceptible to compensatory buffering, whereas Class III and IV agents act on multiple nodes or upstream of the defence systems altogether.

### Chemotherapy and molecular inhibitor-induced ferroptosis

Conventional chemotherapeutic agents remain the cornerstone of systemic cancer treatment by disrupting tumour cell proliferation and survival. However, therapeutic efficacy is frequently limited by the ability of tumour cells to evade regulated cell death, including ferroptosis, thereby promoting chemoresistance [175]. Increasing evidence suggests that modulation of ferroptosis can enhance the antitumour activity of several cytotoxic agents. Cisplatin, an alkylating chemotherapeutic that impairs DNA replication and repair, induces ferroptosis in multiple tumour types, although dysregulation of ferroptosis signalling within the tumour microenvironment (TME) contributes to the development of resistance. Consequently, therapeutic strategies that restore ferroptosis sensitivity have emerged as promising approaches for overcoming cisplatin resistance. For example, eicosapentaenoic acid (EPA) enhances cisplatin-induced ferroptosis in osteosarcoma by downregulating GPX4 expression. In addition to directly promoting ferroptosis, EPA increases tumour-infiltrating CD8⁺ T cells and reverses cisplatin-induced PD-L1 upregulation, thereby strengthening antitumour immune responses [176]. Beyond its direct cytotoxic effects, chemotherapy-induced ferroptosis also remodels the tumour immune microenvironment. In lung cancer, cisplatin promotes ferroptosis while inducing N1 neutrophil polarisation, increasing T-cell infiltration and T helper 1 (Th1) differentiation, thereby converting immunologically “cold” tumours into “hot” tumours. Furthermore, cisplatin and paclitaxel stimulate cancer-associated fibroblasts (CAFs) to secrete exosomal miR-522, which modulates ferroptosis sensitivity and influences the chemosensitivity of gastric cancer cells. Collectively, these findings demonstrate that ferroptosis not only potentiates the cytotoxic effects of chemotherapy but also reshapes antitumour immunity within the TME, supporting the development of ferroptosis-based combination strategies to overcome chemoresistance and improve therapeutic outcomes [141, 177] (Table 1).

**Table 1:** Agents with reported ferroptosis-inducing activity and their current status in oncology clinical trials, classified by mechanism of ferroptosis induction

Agent	FIN class	Primary target	Preclinical evidence	Registered oncology trials (indication; identifier)	Oncology phase	Regulatory status	Ref
Class I - System Xc⁻ inhibition / GSH depletion							
Sorafenib	I	SLC7A11	HCC, GC, ccRCC	HCC (NCT03794440); AML (NCT03247088); neuroblastoma (NCT02559778); lung cancer (NCT00064350)	Approved	Approved	[118]
Sulfasalazine	I	SLC7A11	Prostate cancer, lymphoma, lung cancer, CRC, HNC, PDAC, OCCC	GBM (NCT04205357, NCT01577966); breast cancer (NCT03847311); solid tumours (NCT01198145)	Phase I	Approved (anti-inflammatory)	[127]
Cisplatin	I	GSH depletion	GC, HNC, NSCLC	NSCLC (NCT01656551); bladder cancer (NCT04574960); cervical cancer (NCT01561586); pancreatic cancer (NCT03649321)	Approved	Approved	[176]
Buthionine sulfoximine (BSO)	I	GCL	TNBC	Neuroblastoma (NCT00005835, NCT00002730)	Phase I	Investigational	[30, 139]
Auranofin	I	TrxR1/TrxR2	CLL, ovarian cancer, lung cancer, B-cell lymphoma	CLL (NCT01419691); recurrent epithelial ovarian cancer (NCT01747798); ovarian cancer with sirolimus (NCT03456700); GBM (NCT02770378); lung cancer (NCT01737502)	Phase II	Approved (rheumatoid arthritis)	[178]
Metformin	I	SLC7A11	Breast cancer	Solid tumours with ascorbate (NCT04033107)	Phase II	Approved (type 2 diabetes)	[134]
Class II - Direct GPX4 inhibition							
Gemcitabine	II	GPX4	PDAC, LUAD	Pancreatic cancer (NCT06015659); BTC (NCT05357196); solid tumours (NCT05147272)	Approved	Approved	[179]
Withaferin A	II	GPX4	Neuroblastoma, HCC	Ovarian cancer (NCT05610735); advanced cancer (NCT04092647); osteosarcoma (NCT00689195)	Phase II	Investigational	[139]
Class III - GPX4 degradation with CoQ₁₀ depletion							
Lovastatin	III	HMGCR	NSCLC	Prostate cancer (NCT00580970); ovarian cancer (NCT00585052)		Approved (lipid-lowering)	[139]
Simvastatin	III	HMGCR	TNBC	Multiple myeloma (NCT00281476)	Phase II	Approved (lipid-lowering)	[134]
Class IV - Direct initiation of lipid peroxidation							
Lapatinib	IV	Labile iron ↑	Breast cancer	Breast cancer (NCT03085368, NCT00356811, NCT00667251)	Approved	Approved	[134]
Neratinib	IV	Labile iron ↑	Breast cancer	Breast cancer (NCT04366713); CRC (NCT03377387, NCT03457896)	Approved	Approved	[133]
Artesunate	IV	Labile iron	NHL, HCC	Breast cancer (NCT00764036); CRC (NCT03093129)	Phase II	Approved (antimalarial)	[180]
Pharmacological ascorbate	IV	H₂O₂ generation; labile iron	CRC, PDAC, GBM	GBM (NCT02344355); advanced pancreatic cancer (NCT01515046); metastatic CRC (NCT04516681)	Phase III	Approved (nutritional supplementation)	[137]
Curcumenol	IV	FTH1	Lung cancer	Cancer (NCT00475683)	Phase III	Investigational	[139]
Other mechanisms							
Brequinar		DHODH	Cervical cancer, colon cancer, fibrosarcoma, lung cancer	AML (NCT03760666)	Phase II	Investigational	[31]
β-Elemene		TFEB	NSCLC	NSCLC (NCT03123484); GBM (NCT02629757)	Approved	Approved (China)	[121]

Beyond conventional chemotherapeutic agents, targeted inhibitors have emerged as promising modulators of ferroptosis, offering new opportunities to enhance anticancer immunity and overcome therapeutic resistance. In hepatocellular carcinoma (HCC), inhibition of phosphoglycerate mutase 1 (PGAM1) with the allosteric inhibitor KH3 promotes ferroptosis, increases tumour-infiltrating CD8⁺ T cells, and significantly improves the efficacy of anti-PD-1 immunotherapy. Likewise, protein arginine methyltransferase 5 (PRMT5), an epigenetic regulator implicated in tumour progression, mediates ferroptosis resistance in triple-negative breast cancer (TNBC), whereas pharmacological inhibition with GSK3326595 effectively restores ferroptosis sensitivity [181]. Targeting antioxidant defence pathways has also shown considerable therapeutic potential. The thioredoxin reductase (TrxR) inhibitor auranofin restores responsiveness to anti-PD-1 therapy in head and neck cancer with elevated TrxR expression. Similarly, the leukaemia inhibitory factor receptor (LIFR) inhibitor EC359 promotes ferroptosis in ovarian cancer, enhances leukocyte infiltration, and favours M1 macrophage polarisation, while the FSP1 inhibitor iFSP1 induces tumour ferroptosis, increases immune cell infiltration, and potentiates immune checkpoint blockade in HCC [178]. Modulation of oncogenic signalling pathways further enhances ferroptosis-mediated immunotherapy. Inhibition of the Wnt/β-catenin pathway by ICG001 sensitises melanoma cells to ferroptosis and improves the efficacy of PD-1 blockade, whereas dual PI3K/HDAC inhibition simultaneously induces ferroptosis, remodels the tumour microenvironment towards a pro-inflammatory phenotype, and augments anti-PD-1 responses [182].

Among established ferroptosis inducers, erastin enhances the efficacy of anti-PD-L1 therapy in lung cancer and HCC by inhibiting System Xc⁻ (xCT) and reprogramming tumour-associated macrophages. Likewise, N6F11 selectively degrades GPX4 in tumour cells without affecting immune cells, thereby strengthening antitumour immunity and immune checkpoint blockade. The hybrid compound oxaliplatin–artesunate further exemplifies the therapeutic potential of ferroptosis induction by simultaneously suppressing the glutathione (GSH) defence system, promoting the Fenton reaction and mitochondrial lipid peroxidation, and converting the tumour microenvironment from an immunosuppressive to an immunostimulatory state [139, 183].

### Radiotherapy-induced ferroptosis in the TME

Radiotherapy remains a cornerstone in the management of solid tumours by inducing DNA damage, cell-cycle arrest, senescence, and ultimately tumour cell death. Beyond its direct genotoxic effects, ionising radiation (IR) generates reactive oxygen species (ROS) through water radiolysis and oxidase activation, resulting in oxidative damage to nucleic acids, proteins, and membrane lipids. Increasing evidence indicates that ferroptosis contributes substantially to the therapeutic efficacy of radiotherapy. IR promotes ferroptosis by upregulating acyl-CoA synthetase long-chain family member 4 (ACSL4), thereby enhancing lipid peroxidation. However, tumour cells simultaneously activate adaptive defence mechanisms through increased expression of SLC7A11 and GPX4, limiting ferroptotic cell death and contributing to radioresistance. Consequently, pharmacological induction of ferroptosis has emerged as an effective strategy to sensitise radioresistant tumours to irradiation [20, 184].

Targeting ferroptosis-related pathways further enhances the efficacy of radioimmunotherapy. The selective telomerase inhibitor BIBR1532 increases the radiosensitivity of non-small cell lung cancer by exacerbating IR-induced telomere dysfunction, promoting ROS accumulation, ferroptosis, and mitochondrial stress. Subsequent release of mitochondrial DNA (mtDNA) into the cytoplasm activates the cyclic GMP–AMP synthase–stimulator of interferon genes (cGAS–STING) pathway, triggering an interferon-mediated adaptive immune response and improving the antitumour efficacy of radiotherapy [184, 185].

Ferroptosis also contributes to the radiation-induced bystander effect (RIBE), whereby irradiated cells transmit oxidative and inflammatory signals to neighbouring non-irradiated cells. This process is largely mediated by extracellular vesicles (EVs) and other soluble factors released following irradiation. For example, radiotherapy-derived tumour microparticles (RT-MPs) induce immunogenic ferroptosis, promote M2-to-M1 tumour-associated macrophage (TAM) polarisation, and reverse the immunosuppressive tumour microenvironment. Similarly, RSL3-loaded irradiated tumour cell-derived microparticles (RC@RMPs) effectively induce ferroptosis while remodelling the tumour microenvironment in prostate cancer. Collectively, these findings highlight the considerable potential of combining ferroptosis inducers with radiotherapy to overcome radioresistance, enhance antitumour immunity, and improve therapeutic outcomes [125, 186, 187].

### Nanoparticle-based strategies to induce ferroptosis in the tumour microenvironment

Despite the promising anticancer activity of classical ferroptosis inducers such as erastin and RSL3, their clinical translation remains limited by poor in vivo bioavailability, suboptimal pharmacokinetics, and potential systemic toxicity [1, 20]. Nanotechnology-based delivery systems have emerged as an effective strategy to overcome these limitations by improving drug stability, tumour-specific accumulation, controlled release, and penetration into solid tumours. A wide range of nanoplatforms has therefore been developed as carriers for ferroptosis-targeting therapeutics [188–190].

Beyond drug delivery, ferroptosis-based nanotherapeutics actively remodel the tumour microenvironment (TME). As the immunosuppressive TME is a major driver of therapeutic resistance, recent nanoplatforms have been designed to induce ferroptosis while simultaneously promoting immunogenic cell death (ICD), enhancing dendritic cell (DC) maturation, stimulating cytotoxic CD8⁺ T-cell responses, and improving the efficacy of immune checkpoint blockade. Most of these platforms induce ferroptosis by disrupting the cysteine/glutathione (GSH)/GPX4 antioxidant defence system, thereby amplifying lipid peroxidation and restoring antitumour immunity [76, 191]. An additional advantage of ferroptosis-based nanotherapy is its ability to convert immunologically “cold” tumours into “hot” tumours. Through mechanisms including GPX4 degradation, GSH depletion, hydroxyl radical generation, and Fenton reaction-mediated oxidative stress, nanotherapeutics promote ferroptotic cell death while facilitating dendritic cell activation, T-cell priming, and M2-to-M1 macrophage repolarisation. These coordinated immunometabolic changes reshape the TME, strengthen tumour-specific immune responses, and enhance the effectiveness of immunotherapy [192].

Furthermore, iron-based nanomaterials exploit the catalytic properties of iron to accelerate the Fenton reaction, increase lipid peroxidation, and potentiate ferroptosis. Emerging macrophage-mimetic nanoplatforms further improve therapeutic precision by targeting tumour niches, reprogramming tumour-associated macrophages, and reinforcing ferroptosis-mediated tumour elimination. Although these nano-enabled ferroptosis strategies have produced encouraging preclinical results, their successful clinical translation will depend on further optimisation of biosafety, pharmacokinetics, manufacturing scalability, and validation in well-designed clinical trials [78].

## Ferroptosis and drug resistance

### Tumour Drug Resistance

In oncology, drug resistance refers to the heritable ability of tumour cells to survive exposure to therapeutic concentrations of anticancer agents. Resistance may be primary (intrinsic) or acquired. Primary resistance is present before treatment initiation and commonly arises from genetic mutations, abnormal cellular states, or rapid adaptive responses that render tumour cells inherently insensitive to therapy. In contrast, acquired resistance develops during treatment and represents the predominant cause of therapeutic failure. Two principal mechanisms have been proposed to explain acquired resistance. The pre-existing clone hypothesis suggests that rare drug-resistant subclones are already present before therapy and undergo selective expansion following treatment, ultimately driving tumour recurrence. Alternatively, the re-evolution hypothesis proposes that tumour cells initially survive through epigenetic adaptation and enter a persistent drug-tolerant state before progressively acquiring stable resistance. Beyond tumour-intrinsic mechanisms, the tumour microenvironment (TME) also facilitates therapeutic resistance by providing growth factors and survival signals from tumour and stromal cells, thereby enabling cancer cells to withstand treatment-induced stress [193, 194].

### Mechanisms of tumour drug resistance

The mechanisms underlying tumour resistance are highly complex and multifactorial. As cancer is fundamentally a genetic disease characterised by continuous genetic diversification and clonal evolution, resistance to therapy largely arises from genetic and epigenetic alterations, making both primary and acquired resistance persistent and often irreversible phenomena. Consequently, tumour cells within the same lesion frequently display distinct molecular and phenotypic characteristics, a phenomenon known as tumour heterogeneity [194]. Tumour heterogeneity is a major driver of therapeutic resistance and evolves dynamically during disease progression and treatment, exhibiting both temporal and spatial heterogeneity. This diversity results from genetic and epigenetic alterations within tumour cells and the surrounding tumour microenvironment (TME), including chromosomal instability, missense mutations, DNA methylation, and histone modifications. Such alterations generate resistant subclones that are preferentially selected during therapy, ultimately leading to disease progression and recurrence. Representative examples include chronic myeloid leukaemia (CML), in which BCR-ABL kinase domain mutations confer resistance to imatinib, and non-small cell lung cancer (NSCLC), where multiple subclonal driver mutations, particularly the EGFR T790M mutation, promote resistance to epidermal growth factor receptor (EGFR) tyrosine kinase inhibitors [194, 195].

Disruption of redox homeostasis is a fundamental mechanism underlying tumour drug resistance. Tumour cells adapt to therapeutic stress by limiting reactive oxygen species (ROS) accumulation and enhancing antioxidant capacity, thereby acquiring resistance to multiple anticancer agents [196]. As ferroptosis is intrinsically driven by iron-dependent lipid peroxidation and ROS generation, alterations in cellular redox balance directly influence ferroptosis sensitivity and therapeutic responses. This process represents a dynamic equilibrium in which excessive lipid peroxidation and ROS accumulation promote ferroptotic cell death and suppress tumour progression, whereas enhanced ROS detoxification enables tumour cell survival and facilitates drug resistance [108]. Iron metabolism constitutes another critical determinant of ferroptosis-mediated therapeutic resistance. Dysregulated iron homeostasis, particularly iron overload, promotes ROS generation through the Fenton reaction, supporting tumour growth while simultaneously increasing susceptibility to ferroptosis. However, under therapeutic pressure, tumour cells remodel their metabolic and antioxidant networks to restrict ROS accumulation, thereby escaping ferroptotic cell death and developing resistance. Consistent with this, deferoxamine enhances oxaliplatin sensitivity in cervical cancer, and ferroptosis-associated oxidative stress has been implicated in gemcitabine resistance in pancreatic cancer [197, 198].

Among the antioxidant defence mechanisms, the NRF2 pathway is a master regulator of ferroptosis resistance. Activation of the p62–Keap1–NRF2 signalling axis suppresses erastin- and sorafenib-induced ferroptosis in hepatocellular carcinoma, while persistent Keap1–NRF2 activation in non-small cell lung cancer (NSCLC) promotes resistance to chemotherapy, radiotherapy, and tyrosine kinase inhibitors, and is associated with poor clinical outcomes. Conversely, pharmacological inhibition of NRF2 restores ferroptosis sensitivity and reverses cisplatin resistance in head and neck cancer, whereas excessive NRF2 activation also reduces responsiveness to GPX4 inhibitors. Upstream regulators modulate this axis in opposing directions: iASPP stabilises NRF2 and promotes survival, whereas ARF antagonises NRF2 signalling and enhances ferroptosis susceptibility. NRF2 overexpression additionally confers broad chemoresistance by transcriptionally activating aldo-keto reductases, which eliminate ROS and lipid peroxidation products [1, 2, 72, 109, 199].

### Modulating therapeutic resistance through ferroptosis sensitivity

Given the central role of ferroptosis in multiple biochemical pathways, modulation of its regulatory network has emerged as a promising strategy to overcome tumour drug resistance. Pharmacological induction of ferroptosis enhances the efficacy of conventional anticancer therapies and restores drug sensitivity in resistant tumours. For example, erastin potentiates the antitumour activity of cytarabine and doxorubicin, while ferroptosis induction effectively reverses drug resistance in acute myeloid leukaemia (AML). Several malignancies, including renal cancer, B-cell lymphoma, and triple-negative breast cancer, exhibit marked dependence on GPX4 and System Xc⁻ (xCT) for ferroptosis resistance, highlighting these molecules as attractive therapeutic targets. Likewise, the tumour suppressor p53 promotes ferroptosis by repressing xCT expression, thereby increasing tumour susceptibility to oxidative stress. In addition, non-coding RNAs (ncRNAs) have emerged as important regulators of ferroptosis-mediated drug responses. LncRNA P53RRA modulates ferroptosis through p53, whereas miR-27a and miR-375 restore cisplatin sensitivity by targeting SLC7A11, further supporting ferroptosis-based therapeutic strategies for overcoming treatment resistance [200].

### Ferroptosis and cisplatin resistance

Cisplatin induces both apoptosis and ferroptosis in tumour cells, although the mechanisms responsible for cisplatin resistance predominantly interfere with apoptotic signalling while exerting limited effects on ferroptosis. This distinction makes ferroptosis an attractive strategy for overcoming cisplatin resistance. Pharmacological induction of ferroptosis using erastin markedly enhances cisplatin sensitivity in resistant tumours, particularly in ovarian cancer, where the combination exhibits significant synergistic antitumour activity. Similarly, in cisplatin-resistant gastric cancer, ATF3 cooperates with erastin to suppress the Keap1–NRF2–xCT signalling pathway, thereby promoting ferroptosis and restoring drug sensitivity. In addition, tumours harbouring RAS mutations display increased susceptibility to ferroptosis inducers. Notably, even transient exposure to erastin produces sustained xCT inhibition and glutathione (GSH) depletion, substantially potentiating the cytotoxic effects of cisplatin. Collectively, these findings highlight the considerable therapeutic potential of combining cisplatin with ferroptosis induction to overcome chemoresistance and improve treatment efficacy [1, 10, 40, 201].

### Ferroptosis and resistance to targeted therapy and immunotherapy

Increasing evidence indicates that ferroptosis induction can overcome acquired resistance to multiple targeted therapies, including lapatinib, erlotinib, trametinib, dabrafenib, and vemurafenib. Targeted therapy-resistant tumour cells frequently acquire an epithelial–mesenchymal transition (EMT) phenotype, which promotes therapeutic resistance while simultaneously increasing susceptibility to ferroptosis. Exploiting this vulnerability, β-elemene enhances the sensitivity of KRAS-mutant colorectal cancer cells to cetuximab by inducing ferroptosis and suppressing EMT, highlighting the therapeutic potential of combining ferroptosis induction with targeted therapy [202].

Ferroptosis also plays a pivotal role in determining the efficacy of cancer immunotherapy. Anti-PD-L1 antibodies promote lipid peroxide-dependent ferroptosis in tumour cells, whereas pharmacological inhibition of ferroptosis attenuates their antitumour activity. Moreover, combining immune checkpoint blockade with ferroptosis inducers markedly enhances tumour suppression through interferon-γ (IFN-γ)-mediated activation of STAT1, downregulation of System Xc⁻ (xCT), and subsequent promotion of lipid peroxidation. These findings suggest that ferroptosis induction represents a promising strategy to overcome resistance to both targeted therapy and immunotherapy, thereby improving the durability of anticancer responses [168].

## Clinical translation and clinical trial evidence

Ferroptosis is an attractive therapeutic strategy because tumours that evade apoptosis through TP53 mutation, BCL-2 dysregulation or prolonged chemotherapy exposure remain vulnerable to iron-dependent lipid peroxidation, ferroptotic death occurring largely independently of the caspase machinery. This unique vulnerability provides a compelling rationale for targeting ferroptosis in malignancies that are refractory to conventional chemotherapy and targeted therapies, including sorafenib-resistant hepatocellular carcinoma and platinum-resistant ovarian cancer. Despite this promise, clinical development remains at an early stage. Most available ferroptosis-inducing agents are either repurposed drugs with secondary ferroptotic activity or experimental compounds with limited pharmacokinetic and pharmacodynamic properties, and no therapy has yet received regulatory approval specifically as a ferroptosis inducer [139, 168].

Among currently available agents, sorafenib remains the most clinically advanced compound with recognised ferroptosis-inducing activity. Originally approved as a multikinase inhibitor for hepatocellular carcinoma (HCC) and renal cell carcinoma, sorafenib promotes ferroptosis in part through SLC7A11 inhibition and glutathione depletion. Its clinical application nonetheless preceded recognition of ferroptosis as a therapeutic mechanism, and durable benefit is achieved in only a minority of patients. Acquired resistance arises principally through compensatory antioxidant activation rather than failure of ferroptosis induction, consistent with the network architecture described earlier and indicating the need for combination rather than single-arm targeting [203].

Repurposed drugs with ferroptosis-modulating activity represent the earliest wave of clinical translation, although current evidence remains largely restricted to Phase I/II studies focused on safety and pharmacokinetics rather than therapeutic efficacy. Sulfasalazine, an FDA/EMA-approved System Xc⁻ (xCT) inhibitor, has been investigated as a radiosensitiser in recurrent glioblastoma based on its ability to deplete glutathione (GSH) and enhance radiation-induced oxidative stress. Similarly, artesunate, an antimalarial derivative capable of inducing iron-dependent ferroptosis, has completed Phase I evaluation in advanced solid tumours and metastatic breast cancer, demonstrating acceptable safety profiles but only modest clinical activity. Although nano-liposomal artesunate has shown encouraging preclinical efficacy against hepatocellular carcinoma, clinical validation remains outstanding. Likewise, auranofin, an approved thioredoxin reductase (TrxR) inhibitor, has entered early-phase clinical studies in chronic lymphocytic leukaemia, ovarian cancer, lung cancer, and glioblastoma; however, the limited availability of efficacy data reflects the broader challenge of translating repurposed redox-modulating agents into effective anticancer therapies. Nanomedicine-based platforms show comparable preclinical promise but have not progressed beyond preclinical evaluation [180–205].

Current translational efforts increasingly favour combination over monotherapy. Ferroptosis induction enhances chemotherapy and radiotherapy, as in sulfasalazine combined with temozolomide and stereotactic radiosurgery, and provides a rationale for pairing with immune checkpoint inhibitors, since ferroptotic death releases damage-associated molecular patterns that remodel the tumour immune microenvironment. The network architecture described earlier further implies that combinations should be selected according to which defence arm predominates in a given tumour, rather than pairing inducers empirically [20, 204].

Registered interventional trials evaluating ferroptosis induction as the primary anticancer mechanism nonetheless remain scarce, and the barriers to progress are considered in detail below [15, 20, 206].

The current translational status of the principal ferroptosis-targeting strategies is summarised in Table 2 [42, 54, 79].

**Table 2:** Translational appraisal of ferroptosis-targeting therapeutic strategies in oncology

Therapeutic strategy	Representative agent(s)	Translational readiness	Established to date	Principal evidence gap	Rate-limiting barrier	Ref
System Xc⁻ inhibition (Class I)	Sulfasalazine	Phase I, repurposed	Tolerability established; preclinical radiosensitisation reproducible	No efficacy endpoint reported; ferroptosis engagement unconfirmed in patients	Compensatory transsulfuration flux and alternative cysteine supply	[127]
Multikinase inhibition with incidental ferroptotic activity	Sorafenib	Approved	Clinical benefit in HCC and RCC established; ferroptotic contribution recognised retrospectively	Ferroptosis not a prespecified mechanism in registration trials	Adaptive antioxidant activation, principally NRF2-dependent	[118]
Direct GPX4 inhibition (Class II)	RSL3, ML162	Preclinical only	Potent, reproducible tumour cell killing in vitro and in xenografts	No clinical-stage compound	Poor solubility, short half-life, narrow therapeutic index	[172]
GPX4 degradation with CoQ₁₀ depletion (Class III)	FIN56, statins	Preclinical (FIN56); Phase II (statins)	Dual-node targeting demonstrated preclinically	Statin trials not designed around ferroptotic endpoints	Mevalonate pathway inhibition affects normal tissue equally	[31, 134]
Iron-catalysed peroxidation (Class IV)	Artesunate (oral, IV, nanoliposomal)	Phase II (conventional); preclinical (nanoliposomal)	Safety and pharmacokinetics established; modest single-agent activity	No confirmed activity against ferroptosis-specific endpoints	Iron-rich normal tissues subject to same perturbation	[180]
Pharmacological ascorbate	High-dose intravenous ascorbate	Phase III	Tolerability established across multiple tumour types; KRAS/BRAF-mutant selectivity preclinically	Ferroptotic contribution not separated from other oxidative mechanisms	Mechanistic attribution; pharmacokinetic ceiling	[137]
Thioredoxin reductase inhibition	Auranofin	Phase II, repurposed	Trials completed in CLL and ovarian cancer; tolerability acceptable	No posted efficacy results from completed monotherapy trials	Limited single-agent signal; narrow therapeutic window	[178]
FSP1 inhibition	iFSP1, icFSP1	Preclinical only	Synergy with GPX4 inhibition demonstrated across entities	No clinical-stage compound	Non-canonical vitamin K reductase activity; chemotype-dependent efficacy	[47, 207]
DHODH inhibition	Brequinar	Phase II (non-ferroptotic indication)	Mitochondrial ferroptosis defence targetable; selectivity for GPX4-low tumours	No trial with ferroptotic endpoint or GPX4-stratified population	Requires prospective identification of GPX4-low tumours	[36]
Combination with radiotherapy	Sulfasalazine plus stereotactic radiosurgery	Phase I	Mechanistic rationale strong; ACSL4 upregulation by ionising radiation established	No randomised comparison against radiotherapy alone	Normal-tissue radiosensitisation	[184]
Combination with immune checkpoint blockade	Ferroptosis inducer plus anti-PD-1/PD-L1	Preclinical	IFN-γ-dependent System Xc⁻ suppression and DAMP-mediated immune activation demonstrated	No clinical trial testing the combination prospectively	Ferroptosis of CD8⁺ T cells and NK cells may offset tumour cell killing	[168]
Tumour-selective GPX4 degradation	N6F11	Preclinical	Tumour-restricted degradation sparing immune cells demonstrated in murine models	No clinical-stage compound	Scalability and species-specific selectivity unverified	[150]
Nanomedicine-based induction	Iron oxide and GPX4-inhibitor nanocarriers	Preclinical	Improved tumour accumulation and reduced systemic exposure preclinically	No registered clinical trial	Manufacturing scalability; regulatory pathway; long-term biosafety	[132, 188]

### Challenges and future perspectives

Despite the mechanistic sophistication achieved over the past decade, the clinical translation of ferroptosis-targeting strategies has lagged conspicuously behind the pace of preclinical discovery, a disconnect spanning pharmacology, tumour biology and trial design [193, 194]. The most fundamental obstacle is tumour heterogeneity. Ferroptosis sensitivity is not a fixed cellular property but an emergent state shaped by lineage, metabolic wiring, and microenvironmental context, meaning that a biomarker profile predictive of response in one histology may carry no predictive value in another, and even within a single tumour, subclonal populations can display divergent susceptibility. Compounding this is the context-dependent, at times paradoxical, biology of ferroptosis itself a phenomenon already evident in hepatocellular carcinoma, where ferroptosis can promote malignant transformation from chronic liver injury yet restrain growth once a tumour is established, illustrating that the therapeutic window for ferroptosis induction may shift across disease stages rather than remaining constant. Consequently, future research must shift from indiscriminate reactive oxygen species generation to the precise modulation of membrane lipidomes and iron chaperone networks [208].

A second, arguably more practical barrier is the absence of a validated method to measure ferroptosis in patients. Unlike apoptosis, which can be captured through relatively mature assays (caspase activation, annexin V staining, circulating cleavage fragments), ferroptosis lacks an analogous clinically deployable readout; lipid peroxidation products such as malondialdehyde and 4-HNE are neither specific nor stable enough for routine use, and no imaging or liquid-biopsy platform has been prospectively qualified as a pharmacodynamic marker of in vivo ferroptotic flux [194]. Without such a tool, early-phase trials are effectively blind to whether a candidate agent is engaging its intended mechanism at tolerated doses, which in turn makes it difficult to distinguish genuine mechanistic failure from simple underdosing.

Pharmacological limitations further narrow the translational path. Many of the most potent experimental ferroptosis inducers, RSL3, erastin and related tool compounds, possess poor aqueous solubility, short plasma half-lives, and narrow therapeutic indices that have so far prevented their advancement beyond in vitro and xenograft studies, forcing the field to lean on repurposed drugs such as sorafenib, sulfasalazine, and auranofin, whose ferroptosis activity was discovered incidentally rather than optimised by design. A third and equally fundamental constraint is the therapeutic window. Ferroptosis is not a tumour-restricted process: genetic inactivation of Gpx4 in mice causes lethal acute renal failure, establishing that the same axis targeted therapeutically is indispensable in normal tissue [81]. Liver, kidney and heart are iron-rich, PUFA-rich and metabolically active, and are therefore intrinsically positioned towards ferroptosis competence. Any systemic strategy that lowers antioxidant capacity or raises labile iron is expected to act on these tissues as well as on tumour, and the assumption of tumour selectivity should not be inferred from xenograft efficacy alone.

Selectivity, where it can be achieved, derives from three distinct sources with differing levels of evidence. The first is differential dependency, in which a tumour relies on a defence arm that normal tissue does not require to the same degree. Pancreatic ductal adenocarcinoma is dependent on cystine import through System Xc⁻ to sustain glutathione and coenzyme A synthesis; deletion of Slc7a11 or administration of cyst(e)inase induced tumour-selective ferroptosis and inhibited growth in genetically engineered mice without comparable normal-tissue injury [209]. Clear-cell renal carcinoma shows analogous GPX4 dependence driven by HIF-2α-mediated PUFA enrichment and substantially greater ferroptotic sensitivity than normal renal cells, indicating a genuine window in this lineage [99]. The second is oncogene-driven substrate accumulation. Transformation elevates baseline lipid ROS production, increases transferrin receptor expression and expands the labile iron pool, positioning tumour cells closer to their ferroptotic threshold before treatment begins [81, 209]. Selectivity here is quantitative rather than absolute: the same perturbation is applied to both compartments, but only the tumour is near threshold. This is a narrower and more fragile basis for a window and is likely to be lost where normal tissue is already stressed, as in hepatic injury or reduced renal reserve. The third is pharmacological rather than biological selectivity, achieved through delivery or targeted degradation exemplified by tumour-restricted GPX4 degraders that spare immune cells, and by nanoparticle systems designed to concentrate iron or ferroptosis inducers within tumour tissue [150].

Critically, the first two mechanisms predict that the window is tumour-context-specific rather than a general property of ferroptosis induction. NRF2-hyperactive tumours, which are buffered rather than sensitised, may require perturbations sufficiently intense to exceed normal-tissue tolerance. Conversely, the same reasoning implies that ferroptosis-directed therapy should be prioritised in lineages with demonstrable dependency, such as cystine-addicted or clear-cell tumours, rather than applied indiscriminately. No clinical study to date has prospectively demonstrated a therapeutic window in patients, and organ-function monitoring for hepatic, renal and cardiac toxicity should be regarded as a design requirement rather than a precaution in future trials.

Finally, resistance is not a hypothetical future problem but an already documented one. Tumour cells upregulate compensatory antioxidant nodes (FSP1–CoQ₁₀, GCH1–BH4, DHODH) when the GPX4 axis is pharmacologically suppressed, and the tumour microenvironment itself, through lipid-laden tumour-associated macrophages with impaired ferroptotic-cell clearance, cancer-associated fibroblast-derived cysteine support, and Treg protection by GPX4, actively buffers tumours against ferroptotic stress. Layered onto all of this is simply limited clinical validation: with only a handful of early-phase trials testing repurposed agents and none yet reporting a ferroptosis-specific efficacy endpoint, the field remains short on the randomised, biomarker-stratified evidence needed to move any single strategy forward with confidence.

### Emerging opportunities

Several converging technologies offer plausible routes past these barriers, though none yet resolves them fully. Multiomic profiling integrating transcriptomic, metabolomic and lipidomic layers is beginning to define ferroptosis-susceptibility signatures with greater resolution than single-gene markers, while single-cell and spatial approaches can resolve the subclonal and microenvironmental heterogeneity that bulk sequencing obscures. Patient-derived organoids permit functional testing of inducer sensitivity ex vivo, and genome-scale CRISPR screening continues to reveal druggable regulators; FSP1 itself emerged from such screens [43, 44]. Nanotechnology-based delivery, discussed above, addresses the selectivity and pharmacokinetic limitations of small-molecule inducers.

### Future directions

What is missing is not further mechanistic elaboration but disciplined clinical validation. No ferroptosis biomarker has been prospectively qualified in a clinical trial, no dosing strategy has been benchmarked against a validated pharmacodynamic endpoint, and no randomised comparison has yet tested a ferroptosis-based combination against the standard of care with a prespecified biomarker-defined population. Rectifying this will require trials designed from the outset around patient selection enrolling based on iron-handling and lipid peroxidation phenotype rather than histology alone - paired with combination regimens rather than monotherapy, since the strongest rationale for ferroptosis induction lies in chemosensitisation, radiosensitisation, and synergy with immune checkpoint blockade rather than single-agent cytotoxicity. Treatment-resistant and metastatic disease, where apoptotic pathways are frequently compromised, remain the most biologically plausible niches for ferroptosis-based intervention, but realising this potential depends entirely on solving the biomarker and delivery problems described above rather than on the discovery of additional inducer compounds.

Ferroptosis research has matured from a mechanistic curiosity into a field with genuine, if still largely unrealised, therapeutic promise. Substantial progress has been made in mapping its regulatory architecture and its role in tumour biology and the immune microenvironment, but translating this knowledge into approved therapies will require multidisciplinary collaboration between chemists, oncologists, and biomarker scientists, biomarker-driven patient selection built on prospectively validated assays, rigorous randomised clinical trials rather than continued reliance on repurposed-drug case series, and thoughtful integration with precision oncology frameworks already established for other therapeutic modalities. Achieving this will take years of coordinated effort rather than a single breakthrough compound.

## Conclusion

Ferroptosis has emerged as a pivotal form of regulated cell death with profound implications for cancer biology and therapy. Advances in understanding its molecular regulation have established ferroptosis as a critical determinant of tumour initiation, progression, metastasis, therapeutic resistance, and tumour–immune interactions. The intricate interplay between iron metabolism, lipid peroxidation, antioxidant defence systems, and the tumour microenvironment highlights the complexity of ferroptosis regulation while providing multiple opportunities for therapeutic intervention. Increasing evidence suggests that targeting ferroptosis can enhance the efficacy of chemotherapy, radiotherapy, immunotherapy, and targeted therapy, particularly in treatment-resistant malignancies. Furthermore, emerging approaches such as nanomedicine and rational combination therapies offer promising strategies to improve tumour selectivity and minimise systemic toxicity.

Despite remarkable progress, several challenges continue to impede clinical translation, including tumour heterogeneity, the lack of validated pharmacodynamic biomarkers, limited understanding of context-dependent ferroptosis responses, and concerns regarding off-target toxicity. Future research should prioritise biomarker-guided patient selection, precision medicine approaches, advanced drug-delivery systems, and well-designed clinical trials to fully realise the therapeutic potential of ferroptosis. Collectively, ferroptosis represents a promising therapeutic vulnerability that bridges fundamental cancer biology with translational oncology and is expected to play an increasingly important role in the development of next-generation cancer therapies.

## Data Availability

No datasets were generated or analysed during the current study.

## Abbreviations

AA: Arachidonic acid
ACSL4: Acyl-CoA synthetase long-chain family member 4
AdA: Adrenic acid
AKRs: Aldo-keto reductases
AML: Acute myeloid leukaemia
ATF4: Activating transcription factor 4
BSO: Buthionine sulfoximine
CAFs: Cancer-associated fibroblasts
CLL: Chronic lymphocytic leukaemia
CL: Cardiolipin
CoQ₁₀: Coenzyme Q10
CRC: Colorectal cancer
DAMPs: Damage-associated molecular patterns
DCs: Dendritic cells
DHODH: Dihydroorotate dehydrogenase
EMT: Epithelial–mesenchymal transition
ER: Endoplasmic reticulum
ESCRT: Endosomal sorting complex required for transport
ETC: Electron transport chain
EVs: Extracellular vesicles
FSP1: Ferroptosis suppressor protein 1
FtMt: Mitochondrial ferritin
FTH1: Ferritin heavy chain 1
GCH1: GTP cyclohydrolase 1
GA: Golgi apparatus
GPX4: Glutathione peroxidase 4
GSH: Glutathione
HCC: Hepatocellular carcinoma
HMOX1: Heme oxygenase 1
ICD: Immunogenic cell death
IFN-γ: Interferon gamma
IR: Ionising radiation
LDs: Lipid droplets
LOX: Lipoxygenase
LPO: Lipid peroxidation
LPCAT3: Lysophosphatidylcholine acyltransferase 3
MDA: Malondialdehyde
MDSCs: Myeloid-derived suppressor cells
MFRN: Mitoferrin
mtDNA: Mitochondrial DNA
mtROS: Mitochondrial reactive oxygen species
MVA: Mevalonate
NADPH: Nicotinamide adenine dinucleotide phosphate
ncRNAs: Non-coding RNAs
NCOA4: Nuclear receptor coactivator 4
NK: Natural killer
NRF2: Nuclear factor erythroid 2-related factor 2
NSCLC: Non-small cell lung cancer
OXPHOS: Oxidative phosphorylation
PD-1: Programmed cell death protein 1
PD-L1: Programmed death-ligand 1
PE: Phosphatidylethanolamine
PMN-MDSCs: Polymorphonuclear myeloid-derived suppressor cells
POR: Cytochrome P450 oxidoreductase
PUFAs: Polyunsaturated fatty acids
RCD: Regulated cell death
ROS: Reactive oxygen species
RSL3: RAS-selective lethal 3
SLC7A11: Solute carrier family 7 member 11
SLC3A2: Solute carrier family 3 member 2
STEAP3: Six-transmembrane epithelial antigen of the prostate 3
TAMs: Tumour-associated macrophages
TCA: Tricarboxylic acid
TFRC: Transferrin receptor
TFR1: Transferrin receptor 1
TGF-β: Transforming growth factor-beta
TKIs: Tyrosine kinase inhibitors
TME: Tumour microenvironment
TrxR: Thioredoxin reductase
VDAC: Voltage-dependent anion channel
xCT: Cystine/glutamate antiporter

## References

[1] Zhou Q, Meng Y, Li D, et al. Ferroptosis in cancer: from molecular mechanisms to therapeutic strategies. Signal Transduct Target Ther. 2024;9(1):55–55. 10.1038/s41392-024-01769-5. PMC10920854 38453898

[2] Fu P, Liao M, Qin R, et al. Regulated cell death (RCD) in cancer: key pathways and targeted therapies. Signal Transduct Target Ther. 2022;7(1):286–286. 10.1038/s41392-022-01110-y. PMC9376115 35963853

[3] Koren E, Fuchs Y. Modes of Regulated Cell Death in Cancer. Cancer Discov. 2021;11(2):245–65. 10.1158/2159-8290.cd-20-0789. 33462123

[4] Ke B, Tian M, Li J, Liu B, He G. Targeting Programmed Cell Death Using Small-Molecule Compounds to Improve Potential Cancer Therapy. Med Res Rev. 2016;36(6):983–1035. 10.1002/med.21398. 27357603

[5] Lu J, He R, Liu Y, et al. Exploiting cell death and tumor immunity in cancer therapy: challenges and future directions. Front Cell Dev Biol. 2024;12:1416115–1416115. 10.3389/fcell.2024.1416115. PMC11180757 38887519

[6] Yang WS, Stockwell BR, Ferroptosis. Death by Lipid Peroxidation. Trends Cell Biol. 2015;26(3):165–76. 10.1016/j.tcb.2015.10.014. PMC4764384 26653790

[7] Rochette L, Dogon G, Rigal E, Zeller M, Cottin Y, Vergely C. Lipid Peroxidation and Iron Metabolism: Two Corner Stones in the Homeostasis Control of Ferroptosis. Int J Mol Sci. 2022;24(1):449–449. 10.3390/ijms24010449. PMC9820499 36613888

[8] Diao J, Jia Y, Dai E, et al. Ferroptotic therapy in cancer: benefits, side effects, and risks. Mol Cancer. 2024;23(1):89–89. 10.1186/s12943-024-01999-9. PMC11067110 38702722

[9] Jin X, Tang J, Qiu X, et al. Ferroptosis: Emerging mechanisms, biological function, and therapeutic potential in cancer and inflammation. Cell Death Discov. 2024;10(1):45–45. 10.1038/s41420-024-01825-7. PMC10808233 38267442

[10] Mou Y, Wang J, Wu J, et al. Ferroptosis, a new form of cell death: opportunities and challenges in cancer. J Hematol Oncol. 2019;12(1):34–34. 10.1186/s13045-019-0720-y. PMC6441206 30925886

[11] Riegman M, Sagie L, Galed C, et al. Ferroptosis occurs through an osmotic mechanism and propagates independently of cell rupture. Nat Cell Biol. 2020;22(9):1042–8. 10.1038/s41556-020-0565-1. (PMID: 32868903). PMC7644276 32868903

[12] Hirata Y, Cai R, Volchuk A, et al. Lipid peroxidation increases membrane tension, Piezo1 gating, and cation permeability to execute ferroptosis. Curr Biol. 2023;33(7):1282–94. 10.1016/j.cub.2023.02.060. 36898371

[13] Pedrera L, Espiritu RA, Ros U, et al. Ferroptotic pores induce Ca²⁺ fluxes and ESCRT-III activation to modulate cell death kinetics. Cell Death Differ. 2021;28(5):1644–57. 10.1038/s41418-020-00691-x. PMC8167089 33335287

[14] Feng H, Stockwell BR. Unsolved mysteries: How does lipid peroxidation cause ferroptosis? PLoS Biol. 2018;16(5). 10.1371/journal.pbio.2006203. PMC5991413 29795546

[15] Tang D, Chen X, Kang R, Kroemer G. Ferroptosis: molecular mechanisms and health implications. Cell Res. 2020;31(2):107–25. 10.1038/s41422-020-00441-1. PMC8026611 33268902

[16] Berndt C, Alborzinia H, Amen VS, et al. Ferroptosis in health and disease. Redox Biol. 2024;75:103211–103211. 10.1016/j.redox.2024.103211. PMC11253697 38908072

[17] Bebber CM, Müller F, Clemente LP, Weber J, von Karstedt S. Ferroptosis Cancer Cell Biology Cancers. 2020;12(1):164–164. 10.3390/cancers12010164. PMC7016816 31936571

[18] Hirschhorn T, Stockwell BR. The development of the concept of ferroptosis. Free Radic Biol Med. 2018;133:130–43. 10.1016/j.freeradbiomed.2018.09.043. PMC6368883 30268886

[19] Liu X, Zhang Y, Wu X, et al. Targeting Ferroptosis Pathway to Combat Therapy Resistance and Metastasis of Cancer. Front Pharmacol. 2022;13:909821–909821. 10.3389/fphar.2022.909821. PMC9280276 35847022

[20] Chen Z, Wang W, Razak SRA, Han T, Ahmad NH, Li X. Ferroptosis as a potential target for cancer therapy. Cell Death Dis. 2023;14(7):460–460. 10.1038/s41419-023-05930-w. PMC10366218 37488128

[21] Wang H, Lin D, Yu Q, et al. A Promising Future of Ferroptosis in Tumor Therapy. Front Cell Dev Biol. 2021;9:629150–629150. 10.3389/fcell.2021.629150. PMC8219969 34178977

[22] Bucarey JL, Casas M, Espinosa A. Mitochondrial Iron Handling and Lipid Peroxidation as Drivers of Ferroptosis. Int J Mol Sci. 2026;27(5):2232–2232. 10.3390/ijms27052232. PMC12984849 41828457

[23] Lu Y, Hu J, Chen L, et al. Ferroptosis as an emerging therapeutic target in liver diseases. Front Pharmacol. 2023;14:1196287–1196287. 10.3389/fphar.2023.1196287. PMC10225528 37256232

[24] Zhao L, Zhou X, Xie F, et al. Ferroptosis in cancer and cancer immunotherapy. Cancer Commun. 2022;42(2):88–116. 10.1002/cac2.12250. PMC8822596 35133083

[25] Xiang Y, Song X, Long D. Ferroptosis regulation through Nrf2 and implications for neurodegenerative diseases. Arch Toxicol. 2024;98(3):579–615. 10.1007/s00204-023-03660-8. PMC10861688 38265475

[26] Jyotsana N, Ta KTL, DelGiorno KE. The Role of Cystine/Glutamate Antiporter SLC7A11/xCT in the Pathophysiology of Cancer. Front Oncol. 2022;12. 10.3389/fonc.2022.858462. PMC8904967 35280777

[27] Wu J, Shi Y, Zhou M, et al. Nutrient vitamins enabled metabolic regulation of ferroptosis via reactive oxygen species biology. Front Pharmacol. 2024;15:1434088–1434088. 10.3389/fphar.2024.1434088. PMC11291210 39092216

[28] Li J, Cao F, Yin H, liang, et al. Ferroptosis: past, present and future. Cell Death Dis. 2020;11(2):88–88. 10.1038/s41419-020-2298-2. PMC6997353 32015325

[29] Bai C, Hua J, Meng D, et al. Glutaminase-1 Mediated Glutaminolysis to Glutathione Synthesis Maintains Redox Homeostasis and Modulates Ferroptosis Sensitivity in Cancer Cells. Cell Prolif. 2025;58(11). 10.1111/cpr.70036. PMC12584870 40259435

[30] Cao JY, Dixon SJ. Mechanisms of ferroptosis. Cell Mol Life Sci. 2016;73:2195–209. 10.1007/s00018-016-2194-1. PMC4887533 27048822

[31] Shimada K, Skouta R, Kaplan A, et al. Global survey of cell death mechanisms reveals metabolic regulation of ferroptosis. Nat Chem Biol. 2016;12(7):497–503. 10.1038/nchembio.2079. PMC4920070 27159577

[32] Ding CC, Rose J, Sun T, et al. MESH1 is a cytosolic NADPH phosphatase that regulates ferroptosis. Nat Metab. 2020;2(3):270–7. 10.1038/s42255-020-0181-1. PMC7252213 32462112

[33] Gao M, Monian P, Quadri N, Ramasamy R, Jiang X. Glutaminolysis and transferrin regulate ferroptosis. Mol Cell. 2015;59(2):298–308. 10.1016/j.molcel.2015.06.011 (PMID: 26166707). PMC4506736 26166707

[34] Lee H, Zandkarimi F, Zhang Y, et al. Energy-stress-mediated AMPK activation inhibits ferroptosis. Nat Cell Biol. 2020;22(2):225–34. 10.1038/s41556-020-0461-8. PMC7008777 32029897

[35] Soula M, Weber RA, Zilka O, et al. Metabolic determinants of cancer cell sensitivity to canonical ferroptosis inducers. Nat Chem Biol. 2020;16(12):1351–60. 10.1038/s41589-020-0613-y. PMC8299533 32778843

[36] Mao C, Liu X, Zhang Y, et al. DHODH-mediated ferroptosis defence is a targetable vulnerability in cancer. Nature. 2021;593(7860):586–90. 10.1038/s41586-021-03539-7. PMC8895686 33981038

[37] Hu Y, Huang Y, Zong L, Lin J, Liu X, Ning S. Emerging roles of ferroptosis in pulmonary fibrosis: current perspectives, opportunities and challenges. Cell Death Discov. 2024;10(1):301–301. 10.1038/s41420-024-02078-0. PMC11196712 38914560

[38] Lee S, Hwang N, Seok BG, Lee S, Lee S, Chung SW. Autophagy mediates an amplification loop during ferroptosis. Cell Death Dis. 2023;14(7):464–464. 10.1038/s41419-023-05978-8. PMC10368698 37491375

[39] Wang Y, Hu J, Wu S, et al. Targeting epigenetic and posttranslational modifications regulating ferroptosis for the treatment of diseases. Signal Transduct Target Ther. 2023;8(1):449–449. 10.1038/s41392-023-01720-0. PMC10711040 38072908

[40] Tang Z, Huang Z, Huang Y, et al. Ferroptosis: The Silver Lining of Cancer Therapy. Front Cell Dev Biol. 2021;9:765859–765859. 10.3389/fcell.2021.765859. PMC8667274 34912804

[41] Yang Y, Wang M, Zhang Y, Zhao S, Gu S. The endosomal sorting complex required for transport repairs the membrane to delay cell death. Front Oncol. 2022;12:1007446–1007446. 10.3389/fonc.2022.1007446. PMC9622947 36330465

[42] Sun S, Shen J, Jiang J, Wang F, Min J. Targeting ferroptosis opens new avenues for the development of novel therapeutics. Signal Transduct Target Ther. 2023;8(1):372–372. 10.1038/s41392-023-01606-1. PMC10514338 37735472

[43] Bersuker K, Hendricks JM, Li Z, et al. The CoQ oxidoreductase FSP1 acts parallel to GPX4 to inhibit ferroptosis. Nature. 2019;575(7784):688–92. 10.1038/s41586-019-1705-2. PMC6883167 31634900

[44] Doll S, Freitas FP, Shah R, et al. FSP1 is a glutathione-independent ferroptosis suppressor. Nature. 2019;575(7784):693–8. 10.1038/s41586-019-1707-0. 31634899

[45] Koppula P, Lei G, Zhang Y, et al. A targetable CoQ-FSP1 axis drives ferroptosis- and radiation-resistance in KEAP1 inactive lung cancers. Nat Commun. 2022;13(1):2206. 10.1038/s41467-022-29905-1. PMC9033817 35459868

[46] Higuchi M, Hangauer MJ, Viswanathan VS, et al. FSP1 and histone deacetylases suppress cancer persister cell ferroptosis. Sci Adv. 2026;12(1):eaea8771. 10.1126/sciadv.aea8771. PMC12758554 41481741

[47] Hendricks JM, Doubravsky CE, Wehri E, et al. Identification of structurally diverse FSP1 inhibitors that sensitize cancer cells to ferroptosis. Cell Chem Biol. 2023;30(9):1090–e11037. 10.1016/j.chembiol.2023.04.007. PMC10524360 37178691

[48] Magtanong L, Ko PJ, To M, et al. Exogenous monounsaturated fatty acids promote a ferroptosis-resistant cell state. Cell Chem Biol. 2019;26(3):420–e4329. 10.1016/j.chembiol.2018.11.016. PMC6430697 30686757

[49] Guo J, Zhou Y, Liu D, et al. Mitochondria as multifaceted regulators of ferroptosis. Life Metab. 2022;1(2):134–48. 10.1093/lifemeta/loac035. PMC11749789 39872359

[50] Zhang R, Kroemer G, Tang D. Lipid-derived radical-trapping antioxidants suppress ferroptosis. Life Metab. 2024;3(2). 10.1093/lifemeta/loae008. PMC10960586 38523816

[51] Alves F, Lane DJR, Nguyen TPM, Bush AI, Ayton S. In defence of ferroptosis. Signal Transduct Target Ther. 2025;10(1):2–2. 10.1038/s41392-024-02088-5. PMC11696223 39746918

[52] Battaglia AM, Chirillo R, Aversa I, Sacco A, Costanzo F, Biamonte F. Ferroptosis and Cancer: Mitochondria Meet the Iron Maiden. Cell Death Cells. 2020;9(6):1505–1505. 10.3390/cells9061505. PMC7349567 32575749

[53] Ding Z, Li Z, Sun K, et al. Mitochondrial Regulation of Ferroptosis in Cancer Cells. Int J Biol Sci. 2025;21(5):2179–200. 10.7150/ijbs.105446. PMC11900798 40083691

[54] Yan HF, Zou T, Tuo Q, et al. Ferroptosis: mechanisms and links with diseases. Signal Transduct Target Ther. 2021;6(1):49–49. 10.1038/s41392-020-00428-9. PMC7858612 33536413

[55] Wang Z, Ma J, Li X, et al. Quercetin induces p53-independent cancer cell death through lysosome activation by the transcription factor EB and Reactive Oxygen Species‐dependent ferroptosis. Br J Pharmacol. 2020;178(5):1133–48. 10.1111/bph.15350. 33347603

[56] Chen X, Yu C, Kang R, Tang D. Iron Metabolism in Ferroptosis. Front Cell Dev Biol. 2020;8:590226–590226. 10.3389/fcell.2020.590226. PMC7575751 33117818

[57] Zhu S, Yao R, qi, Li Y, et al. Lysosomal quality control of cell fate: a novel therapeutic target for human diseases. Cell Death Dis. 2020;11(9):817–817. 10.1038/s41419-020-03032-5. PMC7528093 32999282

[58] Torii S, Shintoku R, Kubota C, et al. An essential role for functional lysosomes in ferroptosis of cancer cells. Biochem J. 2016;473(6):769–77. 10.1042/bj20150658. 26759376

[59] Zhang R, Kang R, Tang D. The STING1 network regulates autophagy and cell death. Signal Transduct Target Ther. 2021;6(1):208–208. 10.1038/s41392-021-00613-4. PMC8172903 34078874

[60] Liu Y, Lu SP, Wu L, Yang L, Yang L, Wang J. The diversified role of mitochondria in ferroptosis in cancer. Cell Death Dis. 2023;14(8):519–519. 10.1038/s41419-023-06045-y. PMC10425449 37580393

[61] Gong D, Chen M, Wang Y, Shi J, Hou Y. Role of ferroptosis on tumor progression and immunotherapy. Cell Death Discov. 2022;8(1):427–427. 10.1038/s41420-022-01218-8. PMC9605952 36289191

[62] Zhang Y, Li M, Guo Y, Liu S, Tao Y. The Organelle-Specific Regulations and Epigenetic Regulators in Ferroptosis. Front Pharmacol. 2022;13:905501–905501. 10.3389/fphar.2022.905501. PMC9247141 35784729

[63] Wei J, Seemann J. Golgi ribbon disassembly during mitosis, differentiation and disease progression. Curr Opin Cell Biol. 2017;47:43–51. 10.1016/j.ceb.2017.03.008. PMC5537018 28390244

[64] Mukherjee S, Shields D. Nuclear Import Is Required for the Pro-apoptotic Function of the Golgi Protein p115. J Biol Chem. 2008;284(3):1709–17. 10.1074/jbc.m807263200. PMC2615508 19028683

[65] How PC, Shields D. Tethering Function of the Caspase Cleavage Fragment of Golgi Protein p115 Promotes Apoptosis via a p53-dependent Pathway. J Biol Chem. 2010;286(10):8565–76. 10.1074/jbc.m110.175174. PMC3048739 21147777

[66] Feng Q, Yang Y, Ren K, et al. Broadening horizons: the multifaceted functions of ferroptosis in kidney diseases. Int J Biol Sci. 2023;19(12):3726–43. 10.7150/ijbs.85674. PMC10411478 37564215

[67] Taniguchi M, Nadanaka S, Tanakura S, et al. TFE3 Is a bHLH-ZIP-type Transcription Factor that Regulates the Mammalian Golgi Stress Response. Cell Struct Funct. 2014;40(1):13–30. 10.1247/csf.14015. 25399611

[68] Sassano ML, Tyurina YY, Diokmetzidou A, et al. Endoplasmic reticulum–mitochondria contacts are prime hotspots of phospholipid peroxidation driving ferroptosis. Nat Cell Biol. 2025;27(6):902–17. 10.1038/s41556-025-01668-z. PMC12173944 40514428

[69] Jiang D, Guo Y, Wang T, et al. IRE1α determines ferroptosis sensitivity through regulation of glutathione synthesis. Nat Commun. 2024;15(1):4114–4114. 10.1038/s41467-024-48330-0. PMC11096184 38750057

[70] Wang L, Fang X, Ling B, et al. Research progress on ferroptosis in the pathogenesis and treatment of neurodegenerative diseases. Front Cell Neurosci. 2024;18:1359453–1359453. 10.3389/fncel.2024.1359453. PMC10955106 38515787

[71] Jiang Y, Zhang M, Sun M. ACSL4 at the helm of the lipid peroxidation ship: a deep-sea exploration towards ferroptosis. Front Pharmacol. 2025;16:1594419–1594419. 10.3389/fphar.2025.1594419. PMC12417525 40932861

[72] Nie Z, Chen M, Gao Y, et al. Ferroptosis and Tumor Drug Resistance: Current Status and Major Challenges. Front Pharmacol. 2022;13:879317–879317. 10.3389/fphar.2022.879317. PMC9163417 35668934

[73] Powers SK, Jackson MJ. Exercise-Induced Oxidative Stress: Cellular Mechanisms and Impact on Muscle Force Production. Physiol Rev. 2008;88(4):1243–76. 10.1152/physrev.00031.2007. PMC2909187 18923182

[74] Wu Y, Zhang SW, Gong X, et al. The epigenetic regulators and metabolic changes in ferroptosis-associated cancer progression. Mol Cancer. 2020;19(1):39–39. 10.1186/s12943-020-01157-x. PMC7045519 32103754

[75] Qi D, Peng M. Ferroptosis-mediated immune responses in cancer. Front Immunol. 2023;14:1188365–1188365. 10.3389/fimmu.2023.1188365. PMC10264078 37325669

[76] Wang S, Guo Q, Zhou L, Xia X, Ferroptosis. A double-edged sword. Cell Death Discov. 2024;10(1):265–265. 10.1038/s41420-024-02037-9. PMC11139933 38816377

[77] Wang H, Liu Y, Che S, et al. Deciphering the link: ferroptosis and its role in glioma. Front Immunol. 2024;15:1346585–1346585. 10.3389/fimmu.2024.1346585. PMC10844450 38322268

[78] Shen Z, Song J, Yung BC, Zhou Z, Wu A, Chen X. Emerging Strategies of Cancer Therapy Based on Ferroptosis. Adv Mater. 2018;30(12). 10.1002/adma.201704007. PMC6377162 29356212

[79] Zhang C, Liu X, Jin S, Chen Y, Guo R. Ferroptosis in cancer therapy: a novel approach to reversing drug resistance. Mol Cancer. 2022;21(1):47–47. 10.1186/s12943-022-01530-y. PMC8840702 35151318

[80] Lu B, Chen XB, Ying MD, He Q, Cao J, Yang B. The Role of Ferroptosis in Cancer Development and Treatment Response. Front Pharmacol. 2018;8:992–992. 10.3389/fphar.2017.00992. PMC5770584 29375387

[81] Friedmann Angeli JP, Schneider M, Proneth B, et al. Inactivation of the ferroptosis regulator Gpx4 triggers acute renal failure in mice. Nat Cell Biol. 2014;16(12):1180–91. 10.1038/ncb3064. (PMID: 25402683). PMC4894846 25402683

[82] Dixon SJ, Winter GE, Musavi L, et al. Human Haploid Cell Genetics Reveals Roles for Lipid Metabolism Genes in Nonapoptotic Cell Death. ACS Chem Biol. 2015;10(7):1604–9. 10.1021/acschembio.5b00245. PMC4509420 25965523

[83] Grube J, Woitok MM, Mohs A, et al. ACSL4-dependent ferroptosis does not represent a tumor-suppressive mechanism but ACSL4 rather promotes liver cancer progression. Cell Death Dis. 2022;13(8):704–704. 10.1038/s41419-022-05137-5. PMC9376109 35963845

[84] Chen F, Kang R, Tang D, Liu J. Ferroptosis: principles and significance in health and disease. J Hematol Oncol. 2024;17(1):41–41. 10.1186/s13045-024-01564-3. PMC11157757 38844964

[85] Zeng L, Liu X, Geng C, Gao X, Liu L. Ferroptosis in cancer (Review). Oncol Lett. 2024;28(1):304–304. 10.3892/ol.2024.14437. PMC11106693 38774452

[86] Qi X, Wan Z, Jiang B, et al. Inducing ferroptosis has the potential to overcome therapy resistance in breast cancer. Front Immunol. 2022;13:1038225–1038225. 10.3389/fimmu.2022.1038225. PMC9730886 36505465

[87] Kraft VAN, Bezjian CT, Pfeiffer S, et al. GTP cyclohydrolase 1/tetrahydrobiopterin counteract ferroptosis through lipid remodeling. ACS Cent Sci. 2020;6(1):41–53. 10.1021/acscentsci.9b01063. PMC6978838 31989025

[88] Zou Y, Henry WS, Ricq EL, et al. Plasticity of ether lipids promotes ferroptosis susceptibility and evasion. Nature. 2020;585(7826):603–8. 10.1038/s41586-020-2732-8. PMC8051864 32939090

[89] Timpson P. Faculty Opinions recommendation of Lymph protects metastasizing melanoma cells from ferroptosis. Faculty Opinions – Post-Publication Peer Review of the Biomedical Literature. Published online November. 2020;12. 10.3410/f.738515495.793580094.

[90] Ta N, Jiang X, Zhang Y, Wang H. Ferroptosis as a promising therapeutic strategy for melanoma. Front Pharmacol. 2023;14:1252567–1252567. 10.3389/fphar.2023.1252567. PMC10546212 37795022

[91] Mishima E, Nakamura T, Zheng J, et al. DHODH inhibitors sensitize cancer cells to ferroptosis via FSP1 inhibition. Res Square Preprint posted online Oct. 2022;24. 10.21203/rs.3.rs-2190326/v1.

[92] Mishima E, Ito J, Wu Z, et al. A non-canonical vitamin K cycle is a potent ferroptosis suppressor. Nature. 2022;608(7924):778–83. 10.1038/s41586-022-05022-3. PMC9402432 35922516

[93] Nakamura T, Hipp C, Mourão A, et al. Phase separation of FSP1 promotes ferroptosis. Nature. 2023;619(7969):371–7. 10.1038/s41586-023-06255-6. PMC10338336 37380771

[94] Roeck BF, Vorndran MRH, Basavarajappa L, et al. Ferroptosis spreads to neighboring cells via plasma membrane contacts. Nat Commun. 2025;16(1):2951. 10.1038/s41467-025-58175-w. PMC11947162 40140422

[95] Dixon SJ, Olzmann JA. The cell biology of ferroptosis. Nat Rev Mol Cell Biol. 2024;25(6):424–42. 10.1038/s41580-024-00703-5. (PMID: 38366038). PMC12187608 38366038

[96] Jiang X, Stockwell BR, Conrad M. Ferroptosis: mechanisms, biology and role in disease. Nat Rev Mol Cell Biol. 2021;22(4):266–82. 10.1038/s41580-020-00324-8. PMC8142022 33495651

[97] Liang D, Feng Y, Zandkarimi F et al. Ferroptosis surveillance independent of GPX4 and differentially regulated by sex hormones. Cell. 2023;186(13):2748–2764.e22. 10.1016/j.cell.2023.05.003 (PMID: 37267948). PMC10330611 37267948

[98] Freitas FP, Alborzinia H, Dos Santos AF, et al. 7-Dehydrocholesterol is an endogenous suppressor of ferroptosis. Nature. 2024;626(7998):401–10. 10.1038/s41586-023-06878-9. 38297129

[99] Zou Y, Palte MJ, Deik AA, et al. A GPX4-dependent cancer cell state underlies the clear-cell morphology and confers sensitivity to ferroptosis. Nat Commun. 2019;10(1):1617. 10.1038/s41467-019-09277-9. PMC6453886 30962421

[100] Viswanathan VS, Ryan MJ, Dhruv HD, et al. Dependency of a therapy-resistant state of cancer cells on a lipid peroxidase pathway. Nature. 2017;547(7664):453–7. 10.1038/nature23007. (PMID: 28678785). PMC5667900 28678785

[101] Schwab A, Rao Z, Zhang J, et al. Zeb1 mediates EMT/plasticity-associated ferroptosis sensitivity in cancer cells by regulating lipogenic enzyme expression and phospholipid composition. Nat Cell Biol. 2024;26(9):1470–81. 10.1038/s41556-024-01464-1. PMC11392809 39009641

[102] Hangauer MJ, Viswanathan VS, Ryan MJ, et al. Drug-tolerant persister cancer cells are vulnerable to GPX4 inhibition. Nature. 2017;551(7679):247–50. 10.1038/nature24297. PMC5933935 29088702

[103] Tsoi J, Robert L, Paraiso K, et al. Multi-stage differentiation defines melanoma subtypes with differential vulnerability to drug-induced iron-dependent oxidative stress. Cancer Cell. 2018;33(5):890–e9045. 10.1016/j.ccell.2018.03.017. PMC5953834 29657129

[104] Tan Z, Huang H, Sun W, Li Y, Jia Y. Current progress of ferroptosis study in ovarian cancer. Front Mol Biosci. 2022;9:966007–966007. 10.3389/fmolb.2022.966007. PMC9458863 36090052

[105] Chen R, Cao J, Jiang W, Wang S, Cheng J. Upregulated Expression of CYBRD1 Predicts Poor Prognosis of Patients with Ovarian Cancer. J Oncol. 2021;2021:1–14. 10.1155/2021/7548406. PMC8478559 34594380

[106] Ye L, Wen X, Qin J, et al. Metabolism-regulated ferroptosis in cancer progression and therapy. Cell Death Dis. 2024;15(3):196–196. 10.1038/s41419-024-06584-y. PMC10923903 38459004

[107] Tong X, Tang R, Xiao M, et al. Targeting cell death pathways for cancer therapy: recent developments in necroptosis, pyroptosis, ferroptosis, and cuproptosis research. J Hematol Oncol. 2022;15(1):174–174. 10.1186/s13045-022-01392-3. PMC9733270 36482419

[108] Huang R, Chen H, Liang J, et al. Dual Role of Reactive Oxygen Species and their Application in Cancer Therapy. J Cancer. 2021;12(18):5543–61. 10.7150/jca.54699. PMC8364652 34405016

[109] Li D, Li Y. The interaction between ferroptosis and lipid metabolism in cancer. Signal Transduct Target Ther. 2020;5(1):108–108. 10.1038/s41392-020-00216-5. PMC7327075 32606298

[110] Tang B, Zhu J, Li J, et al. The ferroptosis and iron-metabolism signature robustly predicts clinical diagnosis, prognosis and immune microenvironment for hepatocellular carcinoma. Cell Commun Signal. 2020;18(1):174–174. 10.1186/s12964-020-00663-1. PMC7592541 33115468

[111] Chen Z, Tian H, Yao D, Zhang Y, Feng Z, Yang C. Identification of a Ferroptosis-Related Signature Model Including mRNAs and lncRNAs for Predicting Prognosis and Immune Activity in Hepatocellular Carcinoma. Front Oncol. 2021;11:738477–738477. 10.3389/fonc.2021.738477. PMC8458836 34568075

[112] Wu C, Chen X, Zhang Y, et al. A novel ferroptosis-related signature for predicting prognosis, immune characteristics, and treatment prediction in hepatocellular carcinoma. PLoS ONE. 2025;20(6). 10.1371/journal.pone.0322158. PMC12136417 40465746

[113] Wu X, Cao J, Wan X, Du S. Programmed cell death in hepatocellular carcinoma: mechanisms and therapeutic prospects. Cell Death Discov. 2024;10(1):356–356. 10.1038/s41420-024-02116-x. PMC11310460 39117626

[114] Zheng C, Zhang B, Li Y, et al. Donafenib and GSK-J4 Synergistically Induce Ferroptosis in Liver Cancer by Upregulating HMOX1 Expression. Adv Sci. 2023;10(22). 10.1002/advs.202206798. PMC10401117 37330650

[115] Li L, Wang X, Xu H, Liu X, Xu K. Perspectives and mechanisms for targeting ferroptosis in the treatment of hepatocellular carcinoma. Front Mol Biosci. 2022;9:947208–947208. 10.3389/fmolb.2022.947208. PMC9424770 36052168

[116] Huang Z, Xia H, Cui Y, Yam JWP, Xu Y, Ferroptosis. From Basic Research to Clinical Therapeutics in Hepatocellular Carcinoma. J Clin Transl Hepatol. 2022;0(0):0. 10.14218/jcth.2022.00255. PMC9647096 36406319

[117] Yao F, Deng Y, Zhao Y, et al. A targetable LIFR – NF-κB–LCN2 axis controls liver tumorigenesis and vulnerability to ferroptosis. Nat Commun. 2021;12(1):7333–7333. 10.1038/s41467-021-27452-9. PMC8683481 34921145

[118] Huang W, Chen K, Lu Y, et al. ABCC5 facilitates the acquired resistance of sorafenib through the inhibition of SLC7A11-induced ferroptosis in hepatocellular carcinoma. Neoplasia. 2021;23(12):1227–39. 10.1016/j.neo.2021.11.002. PMC8591347 34768109

[119] Zhang D, Man D, Lu J, et al. Mitochondrial TSPO Promotes Hepatocellular Carcinoma Progression through Ferroptosis Inhibition and Immune Evasion. Adv Sci. 2023;10(15). 10.1002/advs.202206669. PMC10214260 36994647

[120] Zhou Z, Cai Y, Yuan H, et al. Evaluation of Ferroptosis as a Biomarker to Predict Treatment Outcomes of Cancer Immunotherapy. Cancer Res Commun. 2025;5(8):1288–97. 10.1158/2767-9764.crc-25-0268. PMC12326525 40693608

[121] Xu C, Jiang Z, Shao L, et al. β-Elemene enhances erlotinib sensitivity through induction of ferroptosis by upregulating lncRNA H19 in EGFR-mutant non-small cell lung cancer. Pharmacol Res. 2023;191:106739–106739. 10.1016/j.phrs.2023.106739. 36948327

[122] Li Y, Li X, Li J. Ferroptosis in lung cancer: dual role, multi-level regulation, and new therapeutic strategies. Front Oncol. 2024;14:1360638–1360638. 10.3389/fonc.2024.1360638. PMC10955378 38515565

[123] Yang L, Cao L, Zhang X, Chu B. Targeting ferroptosis as a vulnerability in pulmonary diseases. Cell Death Dis. 2022;13(7):649–649. 10.1038/s41419-022-05070-7. PMC9315842 35882850

[124] Xing N, Du Q, Guo S, et al. Ferroptosis in lung cancer: a novel pathway regulating cell death and a promising target for drug therapy. Cell Death Discov. 2023;9(1):110–110. 10.1038/s41420-023-01407-z. PMC10067943 37005430

[125] Maccarinelli F, Coltrini D, Mussi S, et al. Iron supplementation enhances RSL3-induced ferroptosis to treat naïve and prevent castration-resistant prostate cancer. Cell Death Discov. 2023;9(1):81–81. 10.1038/s41420-023-01383-4. PMC9986230 36872341

[126] Cheng XQ, Xu J, Cui Y, et al. Nanovesicles for Lipid Metabolism Reprogram-Enhanced Ferroptosis and Magnetotherapy of Refractory Tumors and Inhibiting Metastasis with Activated Innate Immunity. ACS Nano. 2025;19(7):7213–30. 10.1021/acsnano.4c16981. 39928515

[127] Koppula P, Zhuang L, Gan B. Cystine transporter SLC7A11/xCT in cancer: ferroptosis, nutrient dependency, and cancer therapy. Protein Cell. 2020;12(8):599–620. 10.1007/s13238-020-00789-5. PMC8310547 33000412

[128] Zaffaroni N, Beretta GL. Ferroptosis Inducers for Prostate Cancer Therapy. Curr Med Chem. 2022;29(24):4185–201. 10.2174/0929867329666220111120924. 35021966

[129] Blomme A, Ford CA, Mui E, et al. 2,4-dienoyl-CoA reductase regulates lipid homeostasis in treatment-resistant prostate cancer. Nat Commun. 2020;11(1):2508–2508. 10.1038/s41467-020-16126-7. PMC7237503 32427840

[130] Nassar ZD, Mah CY, Dehairs J, et al. Human DECR1 is an androgen-repressed survival factor that regulates PUFA oxidation to protect prostate tumor cells from ferroptosis. Elife. 2020;9. 10.7554/elife.54166. PMC7386908 32686647

[131] Ai L, Yi N, Qiu C, et al. Revolutionizing breast cancer treatment: Harnessing the related mechanisms and drugs for regulated cell death (Review). Int J Oncol. 2024;64(5). 10.3892/ijo.2024.5634. PMC11000534 38456493

[132] Wang Y, Liu T, Li X, Sheng H, Ma X, Liang H. Ferroptosis-Inducing Nanomedicine for Cancer Therapy. Front Pharmacol. 2021;12. 10.3389/fphar.2021.735965. PMC8722674 34987385

[133] Ren Y, Mao X, Xu H, et al. Ferroptosis and EMT: key targets for combating cancer progression and therapy resistance. Cell Mol Life Sci. 2023;80(9):263–263. 10.1007/s00018-023-04907-4. PMC10439860 37598126

[134] Xu C, Chen Y, Yu Q, Song J, Jin Y, Gao X. Compounds targeting ferroptosis in breast cancer: progress and their therapeutic potential. Front Pharmacol. 2023;14:1243286–1243286. 10.3389/fphar.2023.1243286. PMC10619677 37920209

[135] Morales MJ, Xue X. Targeting iron metabolism in cancer therapy. Theranostics. 2021;11(17):8412–29. 10.7150/thno.59092. PMC8344014 34373750

[136] Ye X, Ju X. Natural products targeting ferroptosis in cancer: molecular mechanisms and applications. Front Oncol. 2025;15:1588668–1588668. 10.3389/fonc.2025.1588668. PMC12454074 40994946

[137] Zhang Y, Xie J. Targeting ferroptosis regulators by natural products in colorectal cancer. Front Pharmacol. 2024;15:1374722–1374722. 10.3389/fphar.2024.1374722. PMC11163120 38860170

[138] Lü L, Chen B, Xu Y, et al. Role of ferroptosis and ferroptosis-related non-coding RNAs in the occurrence and development of gastric cancer. Front Pharmacol. 2022;13:902302–902302. 10.3389/fphar.2022.902302. PMC9421149 36046827

[139] Wang D, Tang L, Zhang Y, et al. Regulatory pathways and drugs associated with ferroptosis in tumors. Cell Death Dis. 2022;13(6):544–544. 10.1038/s41419-022-04927-1. PMC9187756 35688814

[140] Zhang H, Deng T, Liu R, et al. CAF secreted miR-522 suppresses ferroptosis and promotes acquired chemo-resistance in gastric cancer. Mol Cancer. 2020;19(1):43–43. 10.1186/s12943-020-01168-8. PMC7045485 32106859

[141] Yue Z, Yuan Y, Zhou Q, Sheng J, Lin X. Ferroptosis and its current progress in gastric cancer. Front Cell Dev Biol. 2024;12:1289335–1289335. 10.3389/fcell.2024.1289335. PMC10933004 38481532

[142] Dai E, Han L, Liu J, et al. Autophagy-dependent ferroptosis drives tumor-associated macrophage polarization via release and uptake of oncogenic KRAS protein. Autophagy. 2020;16(11):2069–83. 10.1080/15548627.2020.1714209. PMC7595620 31920150

[143] Wu S, Li T, Liu W, Huang Y. Ferroptosis and Cancer: Complex Relationship and Potential Application of Exosomes. Front Cell Dev Biol. 2021;9:733751–733751. 10.3389/fcell.2021.733751. PMC8455874 34568341

[144] Gou Y, Chen M, Li S, et al. Dithiocarbazate-Copper Complexes for Bioimaging and Treatment of Pancreatic Cancer. J Med Chem. 2021;64(9):5485–99. 10.1021/acs.jmedchem.0c01936. 33861929

[145] Yang W, Chi J. Hippo pathway effectors YAP/TAZ as novel determinants of ferroptosis. Mol Cell Oncol. 2019;7(1):1699375–1699375. 10.1080/23723556.2019.1699375. PMC6961671 31993503

[146] Yang W, Ding CC, Sun T, et al. The Hippo Pathway Effector TAZ Regulates Ferroptosis in Renal Cell Carcinoma. Cell Rep. 2019;28(10):2501. 10.1016/j.celrep.2019.07.107. PMC10440760 31484063

[147] Chi H, Li B, Wang Q, et al. Opportunities and challenges related to ferroptosis in glioma and neuroblastoma. Front Oncol. 2023;13:1065994–1065994. 10.3389/fonc.2023.1065994. PMC10021024 36937406

[148] Zhang R, Chen J, Wang S, Zhang W, Zheng Q, Cai R. Ferroptosis Cancer Progression Cells. 2023;12(14):1820–1820. 10.3390/cells12141820. PMC10378139 37508485

[149] Wu J, Minikes AM, Gao M, et al. Intercellular interaction dictates cancer cell ferroptosis via NF2–YAP signalling. Nature. 2019;572(7769):402–6. 10.1038/s41586-019-1426-6. PMC6697195 31341276

[150] Zhou Y, et al. Tumor-specific GPX4 degradation enhances ferroptosis-initiated antitumor immune response in mouse models of pancreatic cancer. Sci Transl Med. 2023. 10.1126/scitranslmed.adg3049. 37910602

[151] Dai E, Han L, Liu J, et al. Ferroptotic damage promotes pancreatic tumorigenesis through a TMEM173/STING-dependent DNA sensor pathway. Nat Commun. 2020;11(1):6339. 10.1038/s41467-020-20154-8. PMC7732843 33311482

[152] Giorgio E, Galeano C, Natali G, et al. Interaction of ferroptosis and immune-mediated inflammation in psoriasis. Antioxid (Basel). 2026;15(3):382. 10.3390/antiox15030382. PMC13023997 41897527

[153] Wang Z, Yang Q, Tan Y, et al. Cancer-Associated Fibroblasts Suppress Cancer Development: The Other Side of the Coin. Front Cell Dev Biol. 2021;9:613534–613534. 10.3389/fcell.2021.613534. PMC7890026 33614646

[154] Wu H, Fu M, Wu M, Cao Z, Zhang Q, Liu Z. Emerging mechanisms and promising approaches in pancreatic cancer metabolism. Cell Death Dis. 2024;15(8):553–553. 10.1038/s41419-024-06930-0. PMC11294586 39090116

[155] Zhu Y, Fang S, Fan B, et al. Cancer-associated fibroblasts reprogram cysteine metabolism to increase tumor resistance to ferroptosis in pancreatic cancer. Theranostics. 2024;14(4):1683–700. 10.7150/thno.89805. PMC10879865 38389839

[156] Su H, Peng C, Liu Y. Regulation of ferroptosis by PI3K/Akt signaling pathway: a promising therapeutic axis in cancer. Front Cell Dev Biol. 2024;12:1372330–1372330. 10.3389/fcell.2024.1372330. PMC10982379 38562143

[157] Clancy J, D’Souza-Schorey C. Tumor-Derived Extracellular Vesicles: Multifunctional Entities in the Tumor Microenvironment. Annu Rev Pathol. 2022;18(1):205–29. 10.1146/annurev-pathmechdis-031521-022116. PMC10410237 36202098

[158] Murray PJ, Wynn TA. Protective and pathogenic functions of macrophage subsets. Nat Rev Immunol. 2011;11(11):723–37. 10.1038/nri3073. PMC3422549 21997792

[159] Chen S, Ma J, Tang J, Yang Y, Zhou S, Feng P. Research progress of macrophage ferroptosis in inflammatory bowel disease and inflammation-cancer transformation. Front Immunol. 2025;16:1658280–1658280. 10.3389/fimmu.2025.1658280. PMC12500433 41063988

[160] Gao W, Wang Y, Zhou Y, Wang X, Yu Y. Autophagy, ferroptosis, pyroptosis, and necroptosis in tumor immunotherapy. Signal Transduct Target Ther. 2022;7(1):196–196. 10.1038/s41392-022-01046-3. PMC9208265 35725836

[161] Li M, He L, Zhu J, Zhang P, Liang S. Targeting tumor-associated macrophages for cancer treatment. Cell Biosci. 2022;12(1):85–85. 10.1186/s13578-022-00823-5. PMC9172100 35672862

[162] Hánělová K, Raudenská M, Masařík M, Balvan J. Protein cargo in extracellular vesicles as the key mediator in the progression of cancer. Cell Commun Signal. 2024;22(1):25–25. 10.1186/s12964-023-01408-6. PMC10777590 38200509

[163] Liu Y, Duan C, Dai R, Zeng Y. Ferroptosis-mediated Crosstalk in the Tumor Microenvironment Implicated in Cancer Progression and Therapy. Front Cell Dev Biol. 2021;9:739392–739392. 10.3389/fcell.2021.739392. PMC8593168 34796174

[164] Zeng W, Zhang R, Huang P, et al. Ferroptotic Neutrophils Induce Immunosuppression and Chemoresistance in Breast Cancer. Cancer Res. 2024;85(3):477–96. 10.1158/0008-5472.can-24-1941. PMC11786957 39531510

[165] Kim R. March. Ferroptosis of tumor neutrophils causes immune suppression in cancer. ScholarlyCommons (University of Pennsylvania). Published online January 1, 2023. Accessed 2026. https://repository.upenn.edu/handle/20.500.14332/59112

[166] Dolina JS, Budimir N, Thomas GD, Salek-Ardakani S. CD8 + T Cell Exhaustion in Cancer. Front Immunol. 2021;12:715234–715234. 10.3389/fimmu.2021.715234. PMC8330547 34354714

[167] Singh S, Mohapatra I, Barik D, et al. Harnessing ferroptosis to transform glioblastoma therapy and surmount treatment resistance. Cell Death Discov. 2025;11(1):448–448. 10.1038/s41420-025-02744-x. PMC12504762 41057317

[168] Zheng X, Jin X, Ye F, et al. Ferroptosis: a novel regulated cell death participating in cellular stress response, radiotherapy, and immunotherapy. Exp Hematol Oncol. 2023;12(1):65–65. 10.1186/s40164-023-00427-w. PMC10375783 37501213

[169] Jin D, Hui Y, Liu D, et al. LINC00942 inhibits ferroptosis and induces the immunosuppression of regulatory T cells by recruiting IGF2BP3/SLC7A11 in hepatocellular carcinoma. Funct Integr Genomics. 2024;24(1):29–29. 10.1007/s10142-024-01292-4. PMC10867055 38353724

[170] Coyle KM, Hawke LG, Ormiston ML. Addressing Natural Killer Cell Dysfunction and Plasticity in Cell-Based Cancer Therapeutics. Cancers. 2023;15(6):1743–1743. 10.3390/cancers15061743. PMC10046032 36980629

[171] Yao L, Hou J, Wu X, et al. Cancer-associated fibroblasts impair the cytotoxic function of NK cells in gastric cancer by inducing ferroptosis via iron regulation. PubMed Cent. 2023;67:102923–102923. 10.1016/j.redox.2023.102923. PMC10582581 37832398

[172] Stockwell BR, Friedmann Angeli JP, Bayir H, et al. Ferroptosis: a regulated cell death nexus linking metabolism, redox biology, and disease. Cell. 2017;171(2):273–85. 10.1016/j.cell.2017.09.021. PMC5685180 28985560

[173] Gaschler MM, Andia AA, Liu H, et al. FINO₂ initiates ferroptosis through GPX4 inactivation and iron oxidation. Nat Chem Biol. 2018;14(5):507–15. 10.1038/s41589-018-0031-6. PMC5899674 29610484

[174] Petriaggi L, Giorgio E, Natali G, et al. Iron fist in a velvet glove: Class IV ferroptosis inducers as a novel strategy to target ovarian cancer. Front Biosci (Landmark Ed). 2025;30(7):39675. 10.31083/FBL39675. 40765348

[175] Singh MK, Arora H, Naik R, et al. Ferroptosis in Cancer: Mechanism and Therapeutic Potential. Int J Mol Sci. 2025;26(8):3852–3852. 10.3390/ijms26083852. PMC12028135 40332483

[176] Liu Q, Wang K. The induction of ferroptosis by impairing STAT3/Nrf2/GPx4 signaling enhances the sensitivity of osteosarcoma cells to cisplatin. Cell Biol Int. 2019;43(11):1245–56. 10.1002/cbin.11121. 30811078

[177] Dong H, Ye C, Yu X, Shao Y. Non-coding RNAs in gastric cancer immunotherapy: mechanisms and clinical implications. Front Immunol. 2025;16:1688181–1688181. 10.3389/fimmu.2025.1688181. PMC12602441 41229433

[178] Tang C, He C, Wang D, et al. Co-delivery of sorafenib and an FSP1 inhibitor triggers dual ferroptosis in tumor cells and immunosuppressive macrophages for enhanced immunotherapy in mouse models of hepatocellular carcinoma. Nat Commun. 2025;16(1):10096–10096. 10.1038/s41467-025-65056-9. PMC12627561 41253833

[179] Yang J, Xu J, Zhang B, et al. Ferroptosis: At the Crossroad of Gemcitabine Resistance and Tumorigenesis in Pancreatic Cancer. Int J Mol Sci. 2021;22(20):10944–10944. 10.3390/ijms222010944. PMC8539929 34681603

[180] Slezáková S, Kučerová J. Anticancer Activity of Artemisinin and its Derivatives. Anticancer Res. 2017;37(11):5995–6003. 10.21873/anticanres.12046. 29061778

[181] Wang Z, Li R, Hou N, et al. PRMT5 reduces immunotherapy efficacy in triple-negative breast cancer by methylating KEAP1 and inhibiting ferroptosis. J Immunother Cancer. 2023;11(6). 10.1136/jitc-2023-006890. PMC10410861 37380368

[182] Fan F, Liu P, Bao R, et al. A Dual PI3K/HDAC Inhibitor Induces Immunogenic Ferroptosis to Potentiate Cancer Immune Checkpoint Therapy. Cancer Res. 2021;81(24):6233–45. 10.1158/0008-5472.can-21-1547. 34711611

[183] Fan R, Deng A, Lin R, et al. A platinum(IV)–artesunate complex triggers ferroptosis by boosting cytoplasmic and mitochondrial lipid peroxidation to enhance tumor immunotherapy. MedComm. 2024;5(6). 10.1002/mco2.570. PMC11106517 38774917

[184] Beretta GL, Zaffaroni N. Radiotherapy-induced ferroptosis for cancer treatment. Front Mol Biosci. 2023;10:1216733–1216733. 10.3389/fmolb.2023.1216733. PMC10304297 37388241

[185] Zhang Z, Liu X, Chen D, Yu J. Radiotherapy combined with immunotherapy: the dawn of cancer treatment. Signal Transduct Target Ther. 2022;7(1):258–258. 10.1038/s41392-022-01102-y. PMC9338328 35906199

[186] Tang H, Cai L, He X, et al. Radiation-induced bystander effect and its clinical implications. Front Oncol. 2023;13:1124412–1124412. 10.3389/fonc.2023.1124412. PMC10113613 37091174

[187] Hu Z, Li H, Gan K, et al. Active-targeting biomimetic nanosystem for prostate cancer enhances radiotherapy efficacy by inducing ferroptosis. J Nanobiotechnol. 2025;24(1):9–9. 10.1186/s12951-025-03879-w. PMC12771893 41318491

[188] Briolay T, Petithomme T, Fouet M, Nguyen-Pham N, Blanquart C, Boisgerault N. Delivery of cancer therapies by synthetic and bio-inspired nanovectors. Mol Cancer. 2021;20(1):55–55. 10.1186/s12943-021-01346-2. PMC7987750 33761944

[189] Biffi S, Voltan R, Bortot B, Zauli G, Secchiero P. Actively targeted nanocarriers for drug delivery to cancer cells. Expert Opin Drug Deliv. 2019;16(5):481–96. 10.1080/17425247.2019.1604679. 30955393

[190] Yao Y, Zhou Y, Liu L, et al. Nanoparticle-Based Drug Delivery in Cancer Therapy and Its Role in Overcoming Drug Resistance. Front Mol Biosci. 2020;7:193–193. 10.3389/fmolb.2020.00193. PMC7468194 32974385

[191] Yang M, Li J, Gu P, Fan X. The application of nanoparticles in cancer immunotherapy: Targeting tumor microenvironment. Bioact Mater. 2020;6(7):1973–87. 10.1016/j.bioactmat.2020.12.010. PMC7773537 33426371

[192] Tang R, Xu J, Zhang B, et al. Ferroptosis, necroptosis, and pyroptosis in anticancer immunity. J Hematol Oncol. 2020;13(1):110–110. 10.1186/s13045-020-00946-7. PMC7418434 32778143

[193] Klemm F, Joyce JA. Microenvironmental regulation of therapeutic response in cancer. Trends Cell Biol. 2014;25(4):198–213. 10.1016/j.tcb.2014.11.006. PMC5424264 25540894

[194] Chatterjee N, Bivona TG. Polytherapy and Targeted Cancer Drug Resistance. Trends Cancer. 2019;5(3):170–82. 10.1016/j.trecan.2019.02.003. PMC6446041 30898264

[195] Pao W, Miller VA, Politi K, et al. Acquired Resistance of Lung Adenocarcinomas to Gefitinib or Erlotinib Is Associated with a Second Mutation in the EGFR Kinase Domain. PLoS Med. 2005;2(3). 10.1371/journal.pmed.0020073. PMC549606 15737014

[196] Yang Z, Hu X, Liu Y, et al. ROS signaling under metabolic stress: cross-talk between AMPK and AKT pathway. Mol Cancer. 2017;16(1):79–79. 10.1186/s12943-017-0648-1. PMC5390360 28407774

[197] Ren Y, Wang R, Weng S, et al. Multifaceted role of redox pattern in the tumor immune microenvironment regarding autophagy and apoptosis. Mol Cancer. 2023;22(1):130–130. 10.1186/s12943-023-01831-w. PMC10413697 37563639

[198] Liu C, Ren L. Enhanced understanding of the involvement of ferroptosis in tumorigenesis: A review of recent research advancements. Cancer Insight. 2023;3(1):37–48. 10.58567/ci03010001.

[199] Gagliardi M, Cotella D, Santoro C, et al. Aldo-keto reductases protect metastatic melanoma from ER stress-independent ferroptosis. Cell Death Dis. 2019;10(12):902–902. 10.1038/s41419-019-2143-7. PMC6883066 31780644

[200] Luo Y, Huang Q, He B, Liu Y, Huang S, Xiao J. Regulation of ferroptosis by non–coding RNAs in the development and treatment of cancer (Review). Oncol Rep. 2020;45(1):29–48. 10.3892/or.2020.7836. PMC7709825 33155665

[201] Yang L, Zhang X, Wang Z, Li B, Zhu H. Modulation of redox homeostasis: A strategy to overcome cancer drug resistance. Front Pharmacol. 2023;14:1156538–1156538. 10.3389/fphar.2023.1156538. PMC10073466 37033606

[202] Zhang H, Chen N, Ding C, Zhang H, Liu D, Liu S. Ferroptosis and EMT resistance in cancer: a comprehensive review of the interplay. Front Oncol. 2024;14:1344290–1344290. 10.3389/fonc.2024.1344290. PMC10926930 38469234

[203] Jiang Y, Yu Y, Pan Z, Glandorff C, Sun M. Ferroptosis: a new hunter of hepatocellular carcinoma. Cell Death Discov. 2024;10(1):136–136. 10.1038/s41420-024-01863-1. PMC10937674 38480712

[204] Wei XX, Jiang Y, Chenwu F, et al. Synergistic Ferroptosis–Immunotherapy Nanoplatforms: Multidimensional Engineering for Tumor Microenvironment Remodeling and Therapeutic Optimization. Nano Micro Lett. 2025;18(1):56–56. 10.1007/s40820-025-01862-6. PMC12405139 40892295

[205] Liang C, Zhang X, Yang M, Dong X. Recent Progress in Ferroptosis Inducers for Cancer Therapy. Adv Mater. 2019;31(51). 10.1002/adma.201904197. 31595562

[206] Li X, Bo Y, Xue D, Qin L. Ferroptosis-immune crosstalk in cervical cancer: mechanisms and therapeutic implications. Front Immunol. 2025;16:1657905–1657905. 10.3389/fimmu.2025.1657905. PMC12395990 40895556

[207] Xavier da Silva TN, Schulte C, Alves AN, Maric HM, Friedmann Angeli JP. Molecular characterization of AIFM2/FSP1 inhibition by iFSP1-like molecules. Cell Death Dis. 2023;14(4):281. 10.1038/s41419-023-05787-z. PMC10119282 37080964

[208] Shen G, Liu J, Wang Y, Deng Z, Deng F. Ferroptosis in Cancer and Inflammatory Diseases: Mechanisms and Therapeutic Implications. MedComm. 2025;6(9). 10.1002/mco2.70349. PMC12409078 40919133

[209] Badgley MA, Kremer DM, Maurer HC, et al. Cysteine depletion induces pancreatic tumor ferroptosis in mice. Science. 2020;368(6486):85–9. 10.1126/science.aaw9872. (PMID: 32241947). PMC7681911 32241947

